# A simulation study on the antiarrhythmic mechanisms of established agents in myocardial ischemia and infarction

**DOI:** 10.1371/journal.pcbi.1012244

**Published:** 2024-06-25

**Authors:** Qince Li, Zheng Yan, Zhen Wang, Cuiping Liang, Xiqian Wang, Xianghu Wu, Wei Wang, Yongfeng Yuan, Kuanquan Wang

**Affiliations:** 1 School of Computer Science and Technology, Harbin Institute of Technology (HIT), Harbin, China; 2 Zibo Central Hospital, Zibo, Shandong, China; 3 Beijing Institute of Computer Technology and Applications, Beijing, China; University of California San Diego, UNITED STATES

## Abstract

Patients with myocardial ischemia and infarction are at increased risk of arrhythmias, which in turn, can exacerbate the overall risk of mortality. Despite the observed reduction in recurrent arrhythmias through antiarrhythmic drug therapy, the precise mechanisms underlying their effectiveness in treating ischemic heart disease remain unclear. Moreover, there is a lack of specialized drugs designed explicitly for the treatment of myocardial ischemic arrhythmia. This study employs an electrophysiological simulation approach to investigate the potential antiarrhythmic effects and underlying mechanisms of various pharmacological agents in the context of ischemia and myocardial infarction (MI). Based on physiological experimental data, computational models are developed to simulate the effects of a series of pharmacological agents (amiodarone, telmisartan, E-4031, chromanol 293B, and glibenclamide) on cellular electrophysiology and utilized to further evaluate their antiarrhythmic effectiveness during ischemia. On 2D and 3D tissues with multiple pathological conditions, the simulation results indicate that the antiarrhythmic effect of glibenclamide is primarily attributed to the suppression of efflux of potassium ion to facilitate the restitution of [K^+^]_o_, as opposed to recovery of I_KATP_ during myocardial ischemia. This discovery implies that, during acute cardiac ischemia, pro-arrhythmogenic alterations in cardiac tissue’s excitability and conduction properties are more significantly influenced by electrophysiological changes in the depolarization rate, as opposed to variations in the action potential duration (APD). These findings offer specific insights into potentially effective targets for investigating ischemic arrhythmias, providing significant guidance for clinical interventions in acute coronary syndrome.

## Author summary

In acute coronary syndrome, the progression of ischemic, injury, and infarction conditions heightens the risk of ischemic cardiac arrhythmias. Despite the existence of various antiarrhythmic drugs, there remains a scarcity of medications specifically tailored for ischemic cardiac arrhythmias. Moreover, comprehending the diverse drug responses in tissues with multiple ischemic conditions (ischemia 1a, 1b, and infarction) remains incomplete. Therefore, in this study, mathematical models depicting the electrophysiological remodeling induced by different drugs such as amiodarone, telmisartan, E-4031, chromanol 293B, and glibenclamide, are constructed and integrated into multi-scale computational models to assess their antiarrhythmic effects. Simulation results demonstrate the potential of glibenclamide in mitigating ischemic cardiac arrhythmias. It can be attributed to its ability not only to improve the depolarization phase’s membrane potential by inhibiting extracellular potassium accumulation during the ischemic stage, leading to an increased dV/dt_max_, but also to extend the action potential duration (APD) by suppressing I_KATP_. Finally, the application of glibenclamide results in heightened conduction velocity and prolonged APD at the tissue level. Consequently, these effects enlarge the wavelength of excitation waves, ultimately reducing the vulnerable window for reentrant wave and exhibiting an anti-ischemic arrhythmic effect. More importantly, it’s shown that glibenclamide’s inhibition of [K^+^]_o_ yields a more significant impact on antiarrhythmic effects compared to its prolongation of APD. This is partly due to the more pronounced enhancement in the wavelength of excitation waves resulting from CV elevation compared with APD prolongation. Additionally, in tissues with coexisting multiple ischemic conditions, suppressing [K^+^]_o_ reduces the spatial dispersion of CV, especially at the boundaries of different ischemic tissues like ischemia 1b and infarction, facilitating a more uniform excitation wave propagation. This study offers insights into the anti-arrhythmic mechanisms of established agents during acute coronary syndrome and identifies potential targets for novel anti-arrhythmic drug development.

## Introduction

As ischemic heart disease progresses, the ion currents and ion concentrations in cardiomyocytes undergo varying degrees of changes, leading to significant differences in myocardial electrophysiological characteristics. Previous studies have shown that the coexistence of multiple electrophysiological remodeling processes during ischemia can lead to an increased risk of arrhythmia [1,2]. The mechanisms underlying arrhythmias induced by the complex coexistence of multiple pathological states remain unclear, and there are currently no therapeutic drugs specifically designed for such complex pathological conditions. In order to explore the antiarrhythmic effects of drugs, it is crucial to construct computational models capable of simulating the ischemic pathological remodeling process [3]. The simulation studies have extensively explored the electrophysiological remodeling during the 1a stage of ischemia [4–6]. Compared to the 1a stage, the 1b stage of ischemia displays marked intercellular decoupling [7]. Nevertheless, the underlying pathological mechanisms of 1b ischemia remain inadequately understood. There exist only a limited number of computational models focused on human ventricular cells during the ischemia 1b phase [2,8]. Furthermore, there is currently a dearth of computational models for acute myocardial infarction (AMI) that are based on human ventricular cells as well [2]. During acute ischemic conditions (ischemia 1a, 1b and AMI), the electrophysiological characteristics of individual cells show a decrease in action potential duration (APD) and amplitude (APA), maximum rate of depolarization (dV/dt_max_), as well as an increase in resting potential (RP) [2]. Furthermore, the conduction properties of cardiac tissue are compromised, resulting in a decreased conduction velocity [7,9,10]. In prior work, we have developed and validated electrophysiological models for distinct ischemic stages [2]. In this study, we integrate pharmacological influences of diverse drugs into these models to simulate their antiarrhythmic effects.

Antiarrhythmic drugs are commonly classified into four main categories: sodium channel antagonists, β-receptor antagonists, potassium channel blockers and activators, and calcium channel antagonists [11]. To tackle pathological remodeling during ischemia processes, the following three types of drugs are selected to investigate their effectiveness in suppressing cardiac arrhythmias. First, drugs like amiodarone [12], E-4031 targeting I_Kr_ inhibition [13], and Chromanol 293B targeting I_Ks_ inhibition [14] are chosen to extend cellular APD. Second, Telmisartan, an agonist of I_Na_ [15], is selected to enhance the excitability of cardiac tissue [16]. Third, Glibenclamide, originally developed as a hypoglycemic agent, can inhibit ATP-sensitive potassium channels, and stimulate insulin secretion [17]. Previous investigations have indicated that Glibenclamide can reduce the frequency of ventricular fibrillation during MI [18], decrease the infarct size [19], and markedly diminish the incidence of ventricular premature beats and non-sustained tachycardia during myocardial ischemia [20]. The antiarrhythmic effect of glibenclamide is closely linked to two factors that influence cardiac electrophysiology: the inhibitory effect on I_KATP_ [21–23] and the ability to promote intracellular potassium retention [22].

In this study, based on detailed physiological experimental data, mathematical models are developed to investigate the effects of these drugs on cellular electrophysiology. With the developed model, the therapeutic efficacy of these drugs is evaluated at the single-cell level and in a 2D myocardial tissue with the pathological condition. In order to determine the primary antiarrhythmic factor, the propagation of reentrant waves and the variations in the vulnerable window (VW) are thoroughly investigated while independently and jointly modifying [K^+^]_o_ and I_KATP_ at different levels (1 μM, 10 μM, and 100 μM) of glibenclamide administration. Finally, the proposed antiarrhythmic hypothesis is validated within anatomically detailed and realistic models of 3D human ventricular tissue. In this study, simulation experiments are conducted in a physiologically relevant and translational setting to establish the efficacy of the proposed antiarrhythmic strategies. Our experimental findings have significantly advanced the understanding of the arrhythmogenic mechanism during short-term ischemia and identified a key factor for designing antiarrhythmic drugs. These results have important implications for developing new therapeutic strategies to ischemic heart diseases, which are associated with an increased risk of life-threatening arrhythmias.

## Methods

### Models of single cells, two-dimensional tissues, and three-dimensional tissues

In the current study, the single-cell models previously developed Liang *et al*. [2] for ischemia and short-term myocardial infarction (MI) are employed to simulate the pathological conditions of acute ischemia. Similarly, the 2D and 3D tissue models in this study are designed with similar geometric and parametric settings as those utilized in the prior studies [2]. Specifically, the single-cell simulation experiment utilizes the S1S2 stimulation protocol. Throughout the simulation process, the steady-state condition is attained through the application of 100 S1 stimuli, each with a 1000 ms interval. The intensity of both S1 and S2 stimuli is -86.2 pA/pF, with a stimulation duration of 1 ms. In the computational models of both two-dimensional and three-dimensional tissues, the diffusion coefficient *D* is determined to be 0.154 mm^2^/ms. Besides the reconstruction of ion currents, decoupling between cells during the ischemic 1b and short-term MI stages may result in a reduction in the conduction velocity of excitation waves within tissues. In this study, the value of *D* in the tissue models representing the ischemic 1b and short-term MI stages is decreased by 30% during the simulation process to mimic the reconstruction of intercellular decoupling.

In both two-dimensional and three-dimensional tissue simulations, the stimulus current intensity is set at -120 pA/pF, with a stimulation duration of 2 ms. The two-dimensional ideal tissue comprised 600 × 600 cells, with a spatial resolution of 0.25 mm. The three-dimensional tissue model consists of isotropic domains of 325 × 325 × 425 cells, with a spatial resolution of 0.33 mm. In the two-dimensional ideal tissue, the S1S2 stimulation protocol is applied with 5 cycles of S1 stimuli, each with a period of 1000 ms, to the leftmost three columns of cells to ensure steady-state conditions. When applying S2 stimulation to either the upper left or lower left corner, the size of the S2 stimulation area is 300 × 300 cells. When applying S2 stimulation to the leftmost column, the size of the S2 stimulation area comprises the leftmost three columns of cells. For dynamic stimulation protocols, when applying stimulation in the upper left corner, the stimulation area size is 5×5 cells. In the three-dimensional ventricular tissue, the stimulation area is situated within the endocardial region and encompassed a size of 5 × 5 × 5 cells. An Intel core i7-3930K 64-bit CPU system is used in our simulation, with acceleration facilitated by parallel computing utilizing a GTX Titan Z GPU.

The simulation results indicate that the conduction velocities (CV) in the normal, ischemic 1a, 1b, and short-term myocardial infarction (MI) tissues are 70.5, 48, 42.75, and 25.5 cm/s, respectively [2]. These findings suggest a decrease in CV in each ischemic tissue compared to the normal state, aligning with experimental data [10, 24].

### Computational modeling of drug effects

Base on comprehensive experimental data, the computational modeling of drug effects is developed by accurately fitting the effects of each drug on electrophysiological properties, such as ion channel dynamics and intracellular/extracellular ion concentrations. In particular, the proportional inhibition of individual ion channels induced by various drugs is determined based on Brennan *et al*.’s single channel blockade theory [25], as demonstrated as follows,

$$g_{v}=\frac{1}{1+{\left(D/IC_{50}\right)}^{nH}},$$ (1)

where, g_v_ represents the proportion of ion channel blockade induced by a drug, *D* represents the drug concentration, *IC*_*50*_ represents the drug concentration corresponding to 50% of ion channel blockade, and *nH* represents the Hill coefficient. Consequently, the present simulation study summarizes the effects of Amiodarone, E-4031, Chromanol 293B, and Telmisartan on electrophysiological properties in Table 1.

**Table 1:** Effects of different drugs on electrophysiological properties.

Drugs	Variations	*IC*_*50*_	*nH*	Channel conductance	Sources of data
Amiodarone	I_Kr_	2.80μM	0.91	71%(1μM); 48%(3μM)	Kamiya[12]
	I_Na_	4.84μM	0.76	76%(1μM); 59%(3μM)	Lalevée [26]
	I_NaK_	15.60μM	1.00	94%(1μM); 84%(3μM)	Gray[27]
	I_CaL_	5.80μM	1.00	85%(1μM); 66%(3μM)	Nishimura[28]
	I_NaCa_	3.30μM	1.00	77%(1μM); 52%(3μM)	Watanabe[29]
	I_Ks_	3.84μM	0.63	69%(1μM); 54%(3μM)	Zankov[30]
E-4031	I_Kr_	15.96±0.04nM	0.74±0.05	30%(30nM); 10%(81nM)	McPate[13]
Chromanol 293B	I_Ks_	1.8 μM	0.8	30%(3.5μM); 10%(10.4μM)	Sun[14]
Telmisartan	I_Na_	1.2μM	1.1	180%(5.5μM); 150%(1.2μM); 110%(0.1μM)	Chang[16]

Regarding the administration of glibenclamide, both I_KATP_ and [K^+^]_o_ are altered. Venkatesh *et al*. [22] have shown that at a concentration of 100 μM glibenclamide, the proportion of [K^+^]_o_ decreases by 32%. Therefore, in the simulation, [K^+^]_o_ is decreased from 8 mM to 5.44 mM. Furthermore, the experimental data indicates that I_KATP_ exhibits a decrease within the range of 51% ± 10%, and there is an improvement of 15% in the reduction of APD. To align with the experimental data obtained from the administration of 100 μM glibenclamide, I_KATP_ is reduced by 40% in the simulation, resulting in an appropriate alteration of APD that is consistent with the experimental observation. Similarly, adjustments are made to I_KATP_ and [K^+^]_o_ to reproduce the experimental observations for 10μM and 1μM glibenclamide. The resultant parameter modifications for simulating the effects of glibenclamide are summarized in Table 2.

**Table 2:** Effects of glibenclamide on electrophysiological properties.

Variations	Glibenclamide			Sources of data
	100μM	10μM	1μM	
I_KATP_	↓40%	↓22%	↓12%	Venkatesh[22]
[K^+^]_o_	5.44mM	6.4mM	7.4mM	

## Results

Initially, comprehensive simulations are conducted to investigate the antiarrhythmic efficacy of previously mentioned drugs, using single-cell models for ischemia 1a, 1b, and AMI respectively and tissue models of ischemia 1a stage. Through analysis of the simulation results, the mechanisms underlying ischemic arrhythmias are investigated, resulting in the identification of the most effective antiarrhythmic drug.

### The antiarrhythmic impact of agents targeting APD prolongation

**Fig 1:**
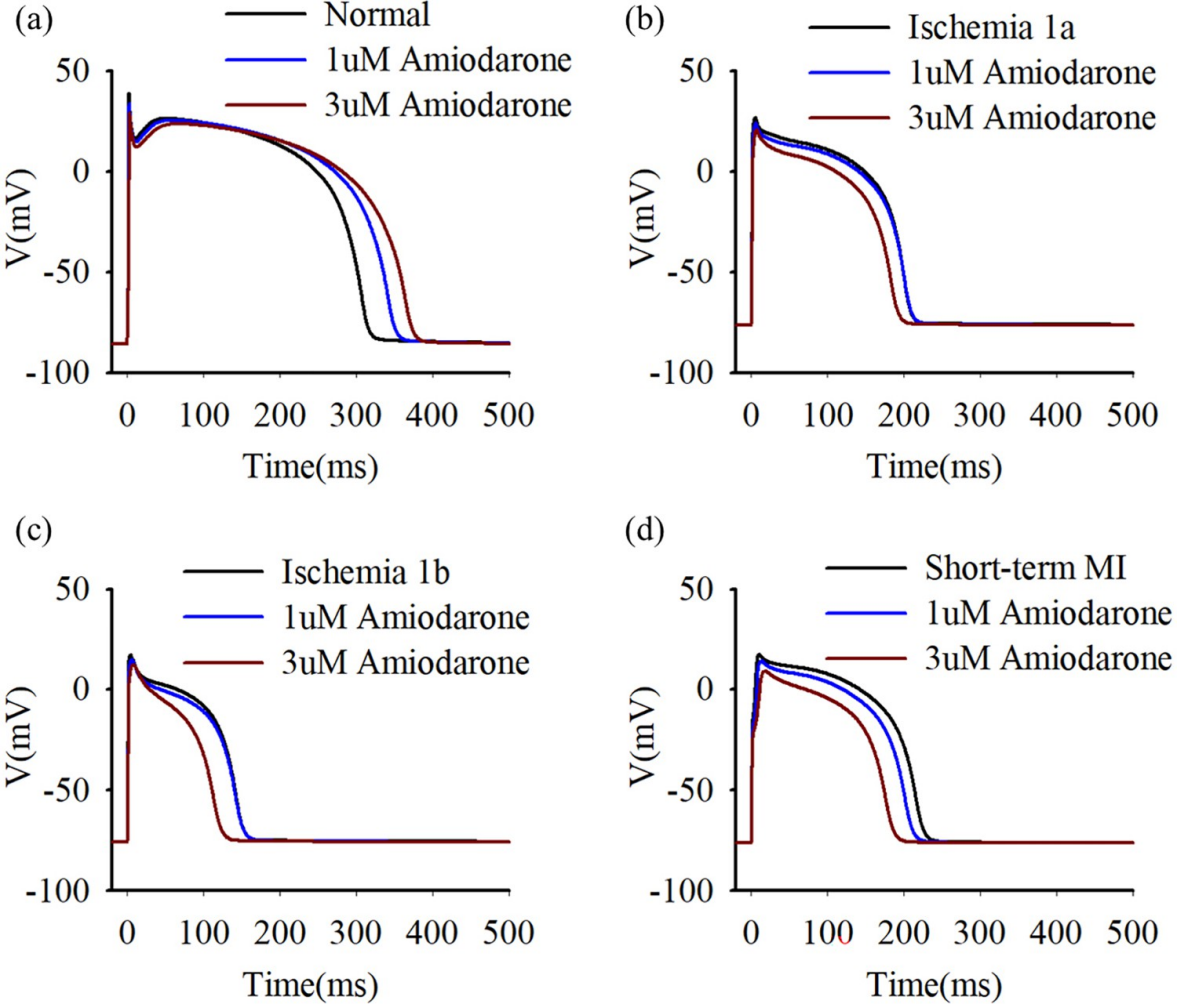
Action potential profiles under normal (a), ischemia 1a (b), ischemia 1b (c), and short-term MI (d) conditions treated with varying dosages of amiodarone. Blue traces represent 1 μM amiodarone, while red traces represent 3 μM amiodarone.

Firstly, we investigate the effects of agents that can prolong APD, including amiodarone, E-4031, and Chromanol 293B. Fig 1 displays the alterations in cellular APD following amiodarone administration. Intriguingly, simulation findings indicate that amiodarone causes a lengthening of APD in normal cardiac cells, whereas it has the contrary effect, causing a further shortening of APD, in cells during the ischemic pathological stages, i.e., ischemia 1a, 1b and AMI. The emergence of this anomalous phenomenon could be attributed to the subsequent mechanisms. In normal cells, the inhibitory effect of amiodarone on K^+^ channels has a pronounced effect on APD, leading to its elongation. Nonetheless, in acute ischemic cells, there’s already a pathological decrease in potassium and L-type calcium currents [2]. Consequently, within ischemic conditions, the further decline in I_Ca_ and I_NaCa_ caused by amiodarone has a more pronounced impact on APD in comparison with K^+^ channel inhibition, eventually resulting in further APD shortening. The simulation results underscore the potential risks associated with administering amiodarone in scenarios where there is remodeling of calcium and potassium channels due to ischemia, emphasizing the need for additional validation through animal and clinical experiments.

**Fig 2:**
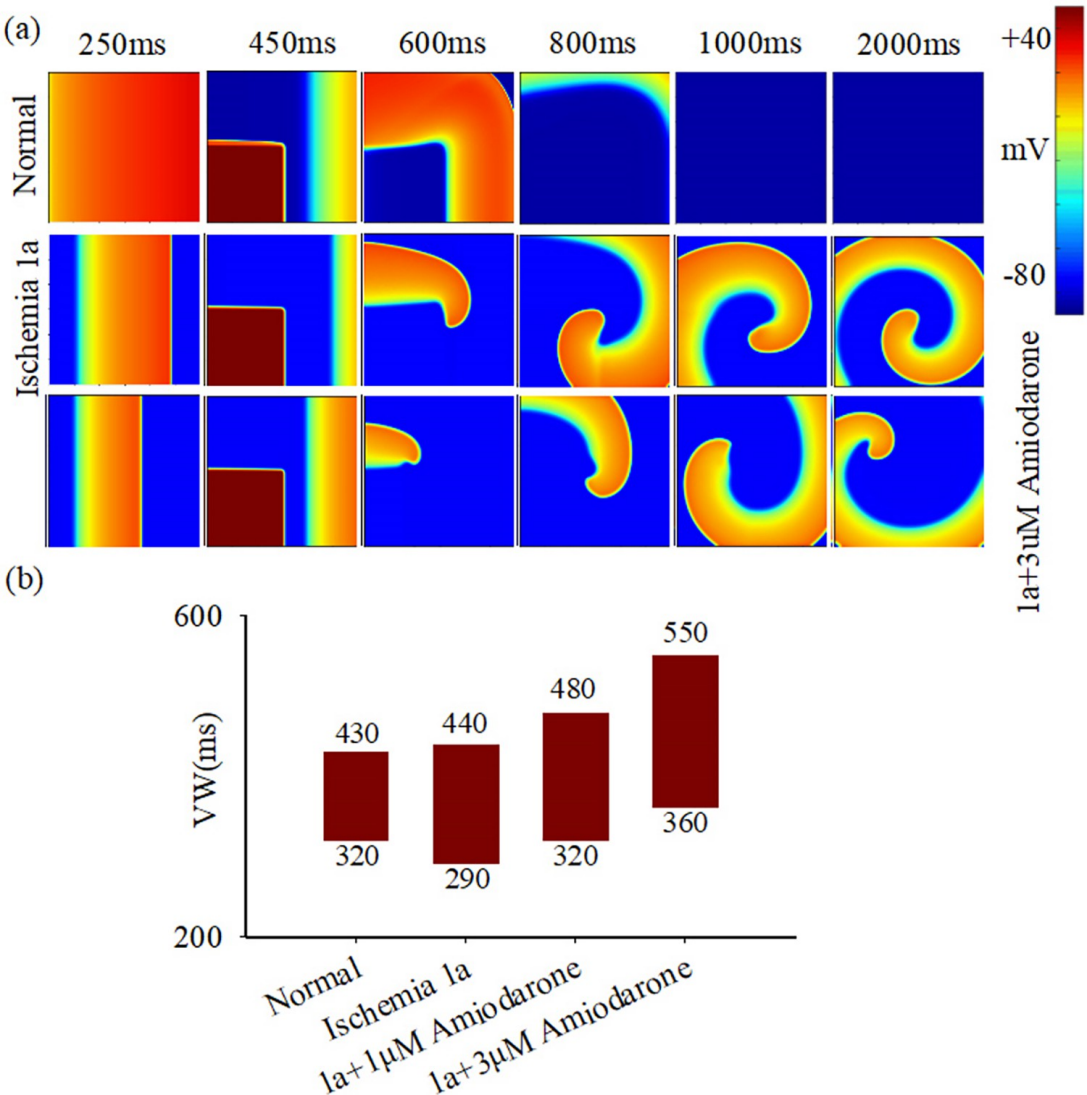
The impact of amiodarone on excitation wave propagation in 2D tissue. (a) Electrical excitation wave patterns under the S1-S2 cross-field stimulation protocol in normal tissue (top row), ischemia 1a tissue before (middle row), and after (bottom row) treatment with 1μM amiodarone. (b) Variations in vulnerable window (VW) in normal and ischemia 1a tissue before and after treatment with 1μM and 3μM amiodarone.

In this study, additional simulations are performed to investigate the effects of amiodarone on pathological tissue of ischemia 1a stage. Fig 2 displays the conduction of electric excitations in normal and ischemic tissue with and without administration of amiodarone. Results demonstrate the absence of reentry waves in normal tissue at a stimulation interval of 450ms, while stable reentry waves are noted in ischemic pathological tissue (ischemia 1a, Fig 2A). Additionally, amiodarone fails to suppress these reentrant waves in ischemic tissue and even augments the vulnerable window (VW) to reentry waves (Fig 2A and 2B). Notably, this phenomenon is observed to be more prominent with an increase in amiodarone dosage. The simulation outcomes at both the single-cell and tissue levels reveal that amiodarone may escalate the vulnerability of ischemic mdyocardial tissue to reentry. This phenomenon can be attributed to amiodarone’s tendency not only to inhibit potassium ion channels but also to affect I_Ca_ and I_NaCa_, ultimately leading to an inability to prolong APD of ischemic cells.

Subsequently, the selective inhibitors of I_Kr_ (E-4031) and I_Ks_ (Chromanol 293B) are employed, both individually and in combination, to evaluate the anti-arrhythmic effects of APD prolongation during acute ischemia (S1 Fig). The simulation results on single cells suggest that E-4031 and Chromanol 293B are principally responsible for augmenting APD. Also, the extent of APD prolongation is found to increase proportionally with the dosage of these drugs (S1A Fig). Notably, the APA and RP remain largely unaffected by these agents, whereas there is a slight decrease in dV/dt_max_ (S1C Fig). Significantly, the combined effect of both drugs is observed to be more pronounced than that of either drug administered individually, as depicted in S1 Fig.

**Fig 3:**
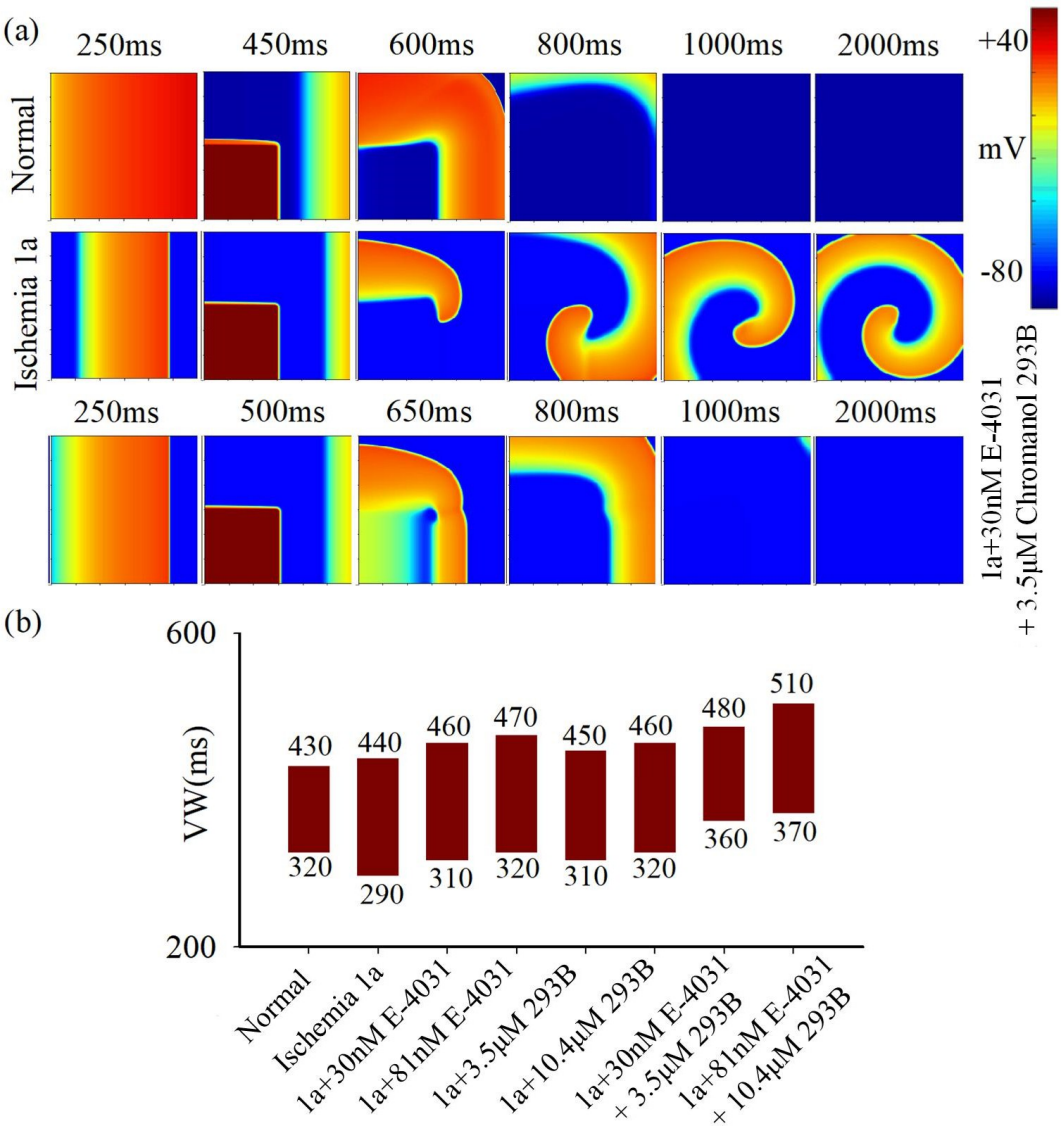
The influence of E-4031 and Chromanol 293B on excitation wave propagation in 2D tissue. (a) Electrical excitation wave patterns under the S1-S2 cross-field stimulation protocol in normal tissue (top row), ischemia 1a tissue before (middle row), and after (bottom row) treatment with 30mM E-4031 and 3.5μM Chromanol 293B. (b) Variations in vulnerable window (VW) in normal and ischemia 1a tissue before and after treatment with 30mM E-4031 and 3.5μM Chromanol 293B individually and in combination.

The therapeutic efficacy of E-4031 and Chromanol 293B is further assessed on 2D myocardial tissue, as depicted in Fig 3. The simulation findings reveal that the administration of both drugs effectively attenuates the development of reentrant waves in ischemic 1a myocardium (Fig 3A). Furthermore, Chromanol 293B modestly reduces the magnitude of the VW when administered alone, while E-4031 does not demonstrate a similar effect. When administered in combination at a low drug concentration, the VW of ischemic 1a myocardium decreases from 150ms to 120ms. However, the VW is 140ms at a high drug concentration (Fig 3B), suggesting a diminished therapeutic impact which may be attributed to the decline in dV/dt_max_ at high drug concentration. In summary, the combined administration of E-4031 and Chromanol 293B can lower the vulnerable window of reentry in myocardial tissue, thereby reducing the risk of arrhythmia for acute ischemic heart disease. However, it should be noted that these two drugs primarily affect the repolarization phase of cells and exert only mild antiarrhythmic effects.

### The antiarrhythmic effect of agents targeting conduction enhancement

**Fig 4:**
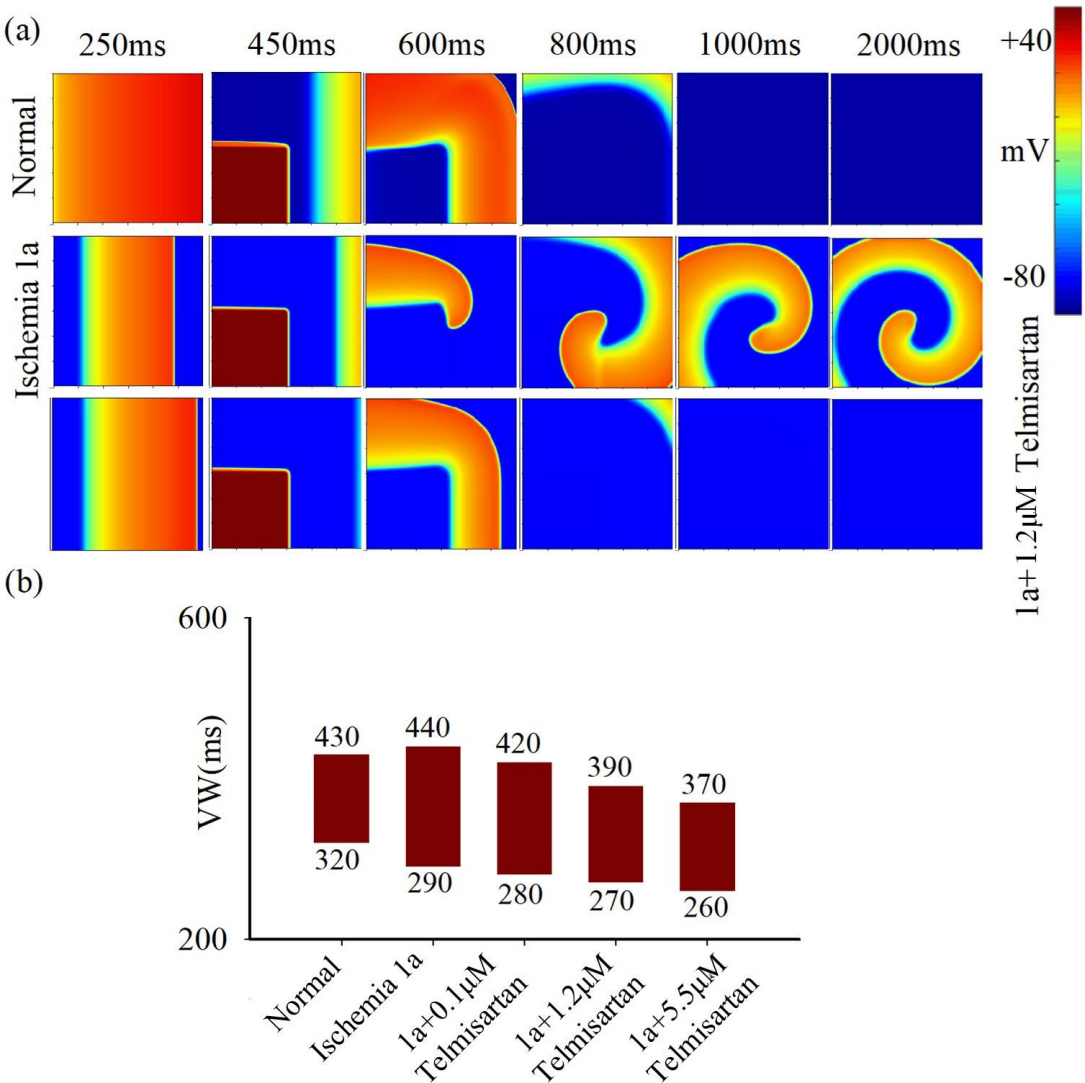
The impact of telmisartan on excitation wave propagation in 2D tissue. (a) Electrical excitation wave patterns under the S1-S2 cross-field stimulation protocol in normal tissue (top row), ischemia 1a tissue before (middle row), and after (bottom row) treatment with 1.2μM telmisartan. (b) Variations in vulnerable window (VW) in normal and ischemia 1a tissue before and after treatment with 0.1μM, 1.2μM and 5.5μM telmisartan.

As an I_Na_ agonist, telmisartan demonstrates the potential to augment the electrical conduction properties of myocardial tissue. As illustrated in S2 Fig, telmisartan elevates dV/dt_max_ during the repolarization phase of ischemic1a cells, consequentially resulting in an escalation of the conduction velocity of electrical excitation waves within the tissue (S2B Fig). However, telmisartan exhibites limited influence on the duration of APD and RP (S2C Fig). These results explicitly suggest that telmisartan has a significant impact on cell depolarization and has minimal impact on cell repolarization. Subsequent simulations are performed on 2D tissue to investigate the propagation of electric excitation waves in pathological tissue of ischemia 1a before and after the administration of telmisartan, as shown in Fig 4. The simulation results demonstrate that telmisartan effectively inhibits the development of re-entry waves in the pathological tissue of ischemic 1a and reduces the magnitude of the VW that facilitates re-entry. Furthermore, this effect is observed to increase with higher doses of telmisartan. By administering a high dose of telmisartan, the VW of ischemic 1a myocardium decreases from 150ms to 110ms.

### The antiarrhythmic effect of the multi-factor targeting drug

**Fig 5:**
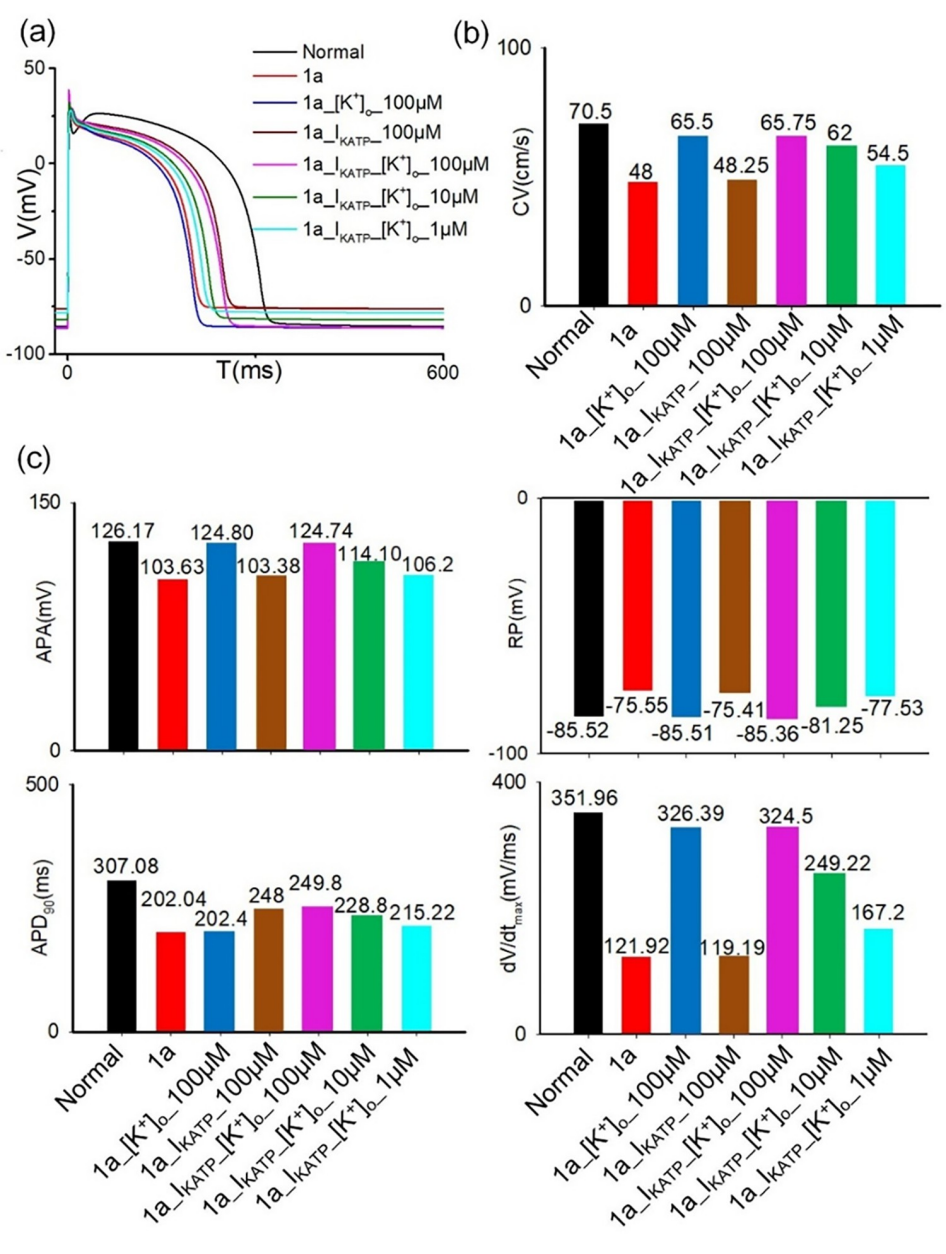
Variations in electrophysiological characteristics of cardiomyocytes under different conditions: normal, ischemia 1a, changing [K^+^]_o_ or I_KATP_ alone at 100μM glibenclamide in ischemia 1a and changing I_KATP_ and [K^+^]_o_ simultaneously at 100μM, 10μM, and 1μM glibenclamide in ischemia 1a. (a)AP profiles (b)CVs (c) AP characteristics, including APA, RP, APD_90_, and dV/dt_max_.

In this study, the antiarrhythmic effects of glibenclamide are investigated by inhibiting I_KATP_ and reducing the extracellular potassium concentration ([K^+^]_o_) independently and jointly. During the ischemia 1a phase, significant alterations are observed in both the depolarization and repolarization phases of the action potential upon administration of glibenclamide. As illustrated in Fig 5, these changes are characterized by a marked prolongation of APD and a decline of RP. When [K^+^]_o_ is solely reduced from 8 mM to 5.44 mM during ischemia 1a, a notable augmentation in the conduction velocity (CV) and dV/dt_max_ is observed (Fig 5B). Thereafter, the CV and dV/d_tmax_ were restored nearly to the baseline level under normal conditions. Moreover, the RP and APA exhibit remarkable recovery nearly to the levels observed under normal physiological conditions. In contrast, the APD remains unaltered (Fig 5C). However, when the I_KATP_ is solely down-regulated by 40% during ischemia 1a, the APD increases from 202.04 ms to 248 ms, without significant changes to other physiological characteristics. The simulation results suggest that alterations in [K^+^]_o_ primarily affect the depolarization phase (such as dV/dt_max_, RP and APA), with little impact on the APD during the development of myocardial ischemia. Conversely, a decrease in I_KATP_ predominantly affects the APD, while exerting minimal effects on the depolarization phase. Furthermore, it is worth noting that magnitude of therapeutic response depends on the dosage of glibenclamide utilized. The therapeutic effect observed at the dose of 100 or 10 μM glibenclamide, is more significant compared to that achieved with a concentration of 1μM glibenclamide (Fig 5).

### The impact of glibenclamide on 2D ischemic cardiac tissues

**Fig 6:**
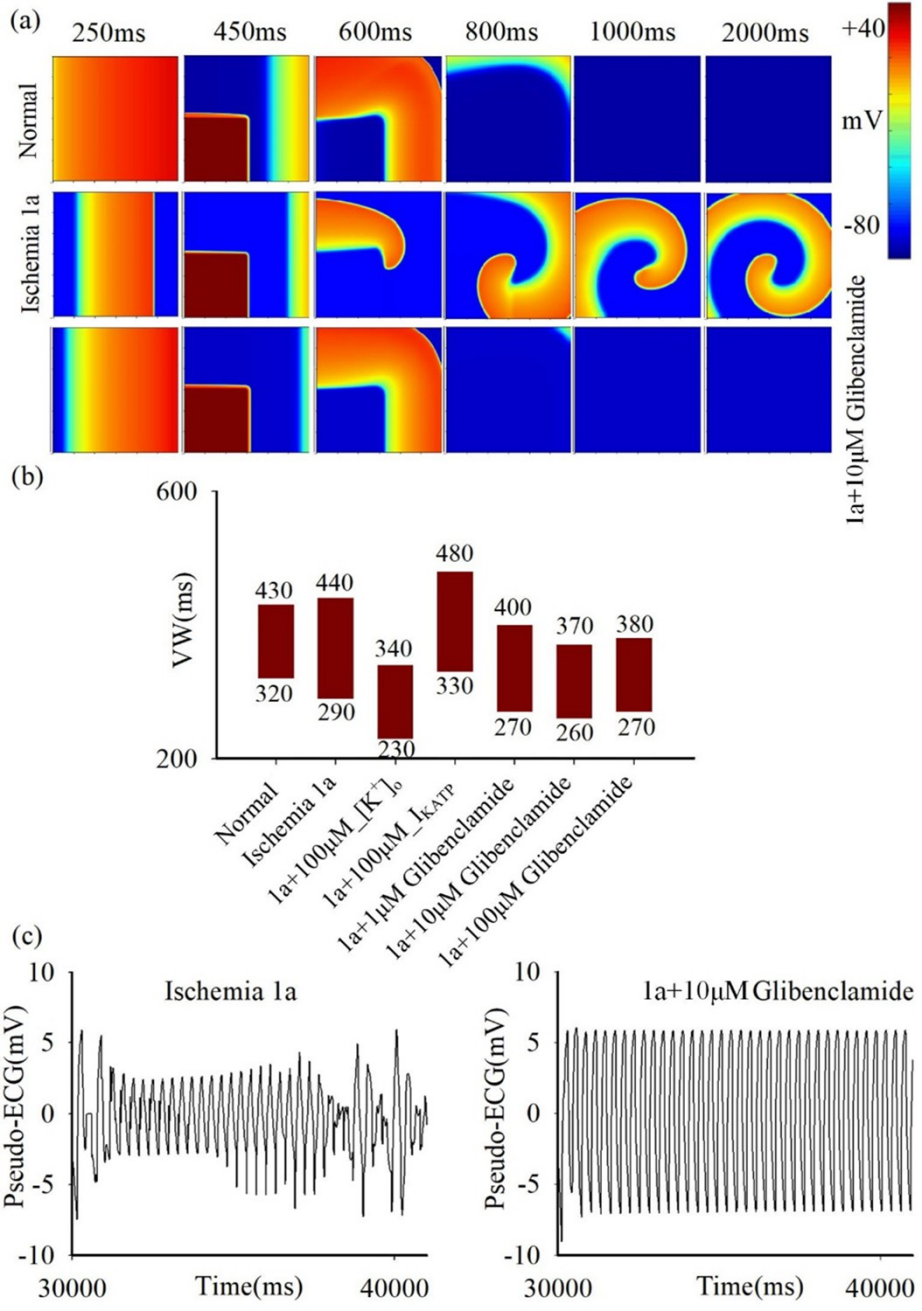
The impact of glibenclamide on excitation wave propagation in 2D homogenous tissue. (a) Electrical excitation wave patterns under the S1-S2 cross-field stimulation protocol in normal tissue (top row), ischemia 1a tissue before (middle row), and after (bottom row) treatment with 10μM glibenclamide. (b) Variations in vulnerable window (VW) in normal and ischemia 1a tissue before and after treatment with 100μM, 10μM and 1μM glibenclamide. In particular, 1a+100μM_[K^+^]_o_ and 1a+100μM_I_KATP_ represent altering [K^+^]_o_ and I_KATP_ individually during the administration of 100μM glibenclamide. (c) Pseudo-ECG (PCL = 300ms) with and without the administration of 10μM glibenclamide in 2D ischemia 1a tissues.

This study further investigates the antiarrhythmic mechanism of glibenclamide by examining its effect on excitation wave propagation in two-dimensional cardiac tissues with a single pathological condition and with multiple pathological conditions. Firstly, the induction of reentrant waves in 2D homogeneous tissues with ischemia 1a is investigated using the S1-S2 cross-field stimulation protocol. Fig 6 illustrates the wave propagation and VWs on the tissue before and after administering glibenclamide treatment. Simulation results indicated glibenclamide treatment is effective in inhibiting the reentry wave generation in the pathological tissue of ischemia 1a (Fig 6A). During the simulation applying glibenclamide, the restoration of [K^+^]_o_ alone (from an original value of 8 mM to 5.44 mM) considerably decreases the width of VW. In contrast, the restoration of only I_KATP_ does not significantly lower the width of VW. Furthermore, both 100 μM and 10 μM concentrations of glibenclamide exhibit comparable antiarrhythmic efficacy, which is superior to that of 1 μM concentration. In particular, the VW is found to be approximate 110 ms for both 100 μM and 10 μM, smaller than that detected at 1 μM (130 ms, Fig 6B). Additionally, the pseudo-ECGs illustrated in Fig 6C (left panel) highlights the manifestation of severe arrhythmias in ischemic 1a tissue with a pacing cycle length of 300ms. The cardiac rhythm is improved significantly upon the administration of 10 μM glibenclamide, underscoring its promising therapeutic potential in the treatment of arrhythmias in ischemic tissue (Fig 6C, right panel). Moreover, the antiarrhythmic efficacy of glibenclamide is similar in 2D homogeneous tissue models characterized by other single pathological conditions, including ischemia 1b or AMI, as depicted in S3 Fig.

**Fig 7:**
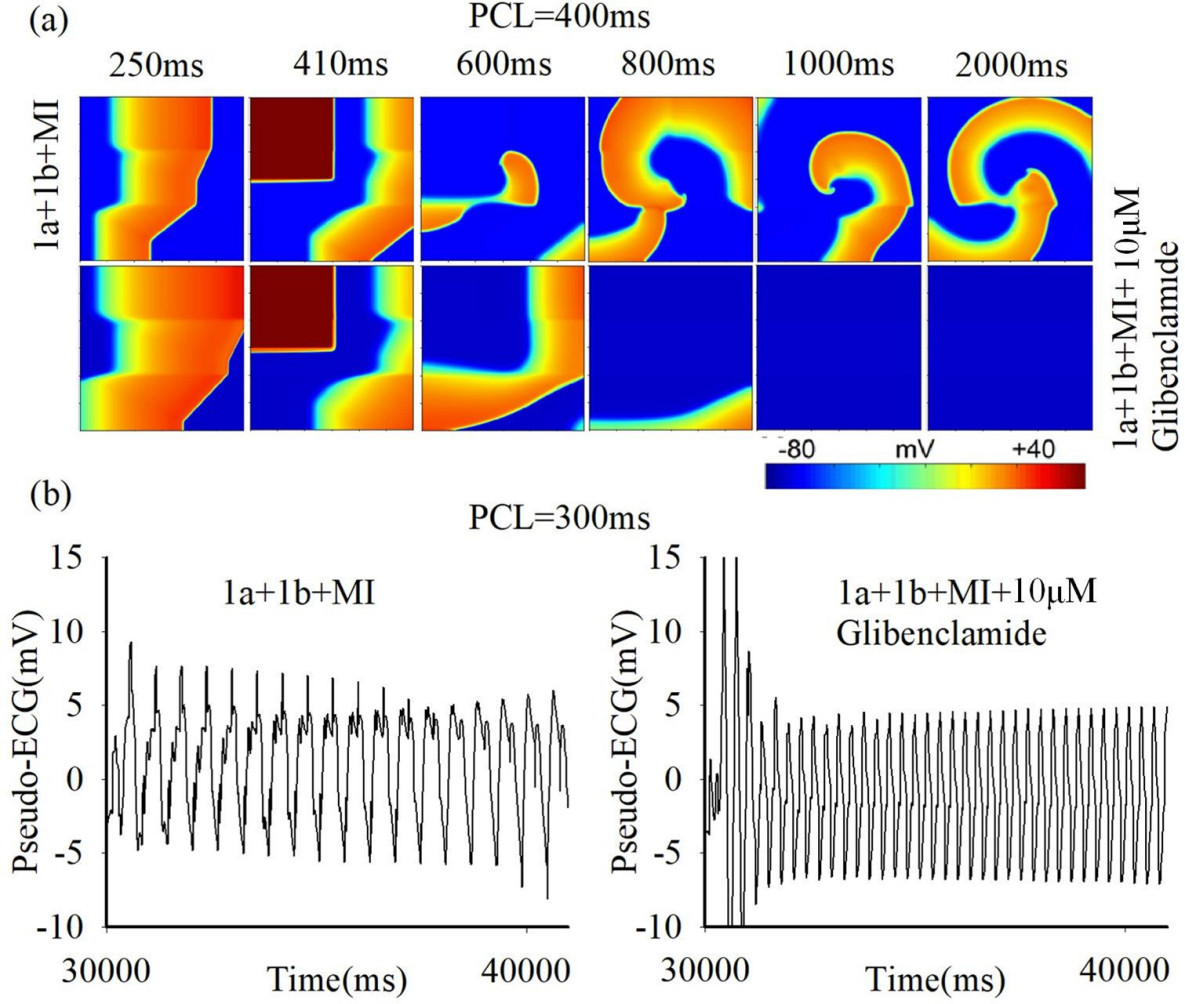
The impact of glibenclamide on excitation wave propagation in 2D heterogeneous tissue with multiple ischemic conditions. (a) Electrical excitation wave patterns under the S1-S2 cross-field stimulation protocol in tissues featuring multiple ischemic conditions with (bottom row) and without (top row) the administration of 10μM glibenclamide. (b) Pseudo-ECG (PCL = 300ms) in these tissues, comparing scenarios with and without the administration of 10μM glibenclamide.

Given that various pathological conditions can coexist in cardiac tissue during the development of ischemia [1,2], the effects of glibenclamide are further evaluated on inhomogeneous tissues with multiple pathological conditions, including ischemia 1a, 1b, and myocardial infarction (MI) regions from top to bottom, as depicted in Fig 7. Furthermore, the excitation waves induced by the S1-S2 stimulation protocol illustrates that the presence of multiple sustained reentrant waves leads to the breakup of these waves (Fig 7A), resulting in highly disordered electrocardiographic signals on the ischemic tissue with multiple pathological conditions (Fig 7B, left panel). The administration of glibenclamide restores regularity to the disordered electrocardiogram (Fig 7B, right panel), indicating its potential as an effective antiarrhythmic agent for tissues with multiple pathological conditions.

**Fig 8:**
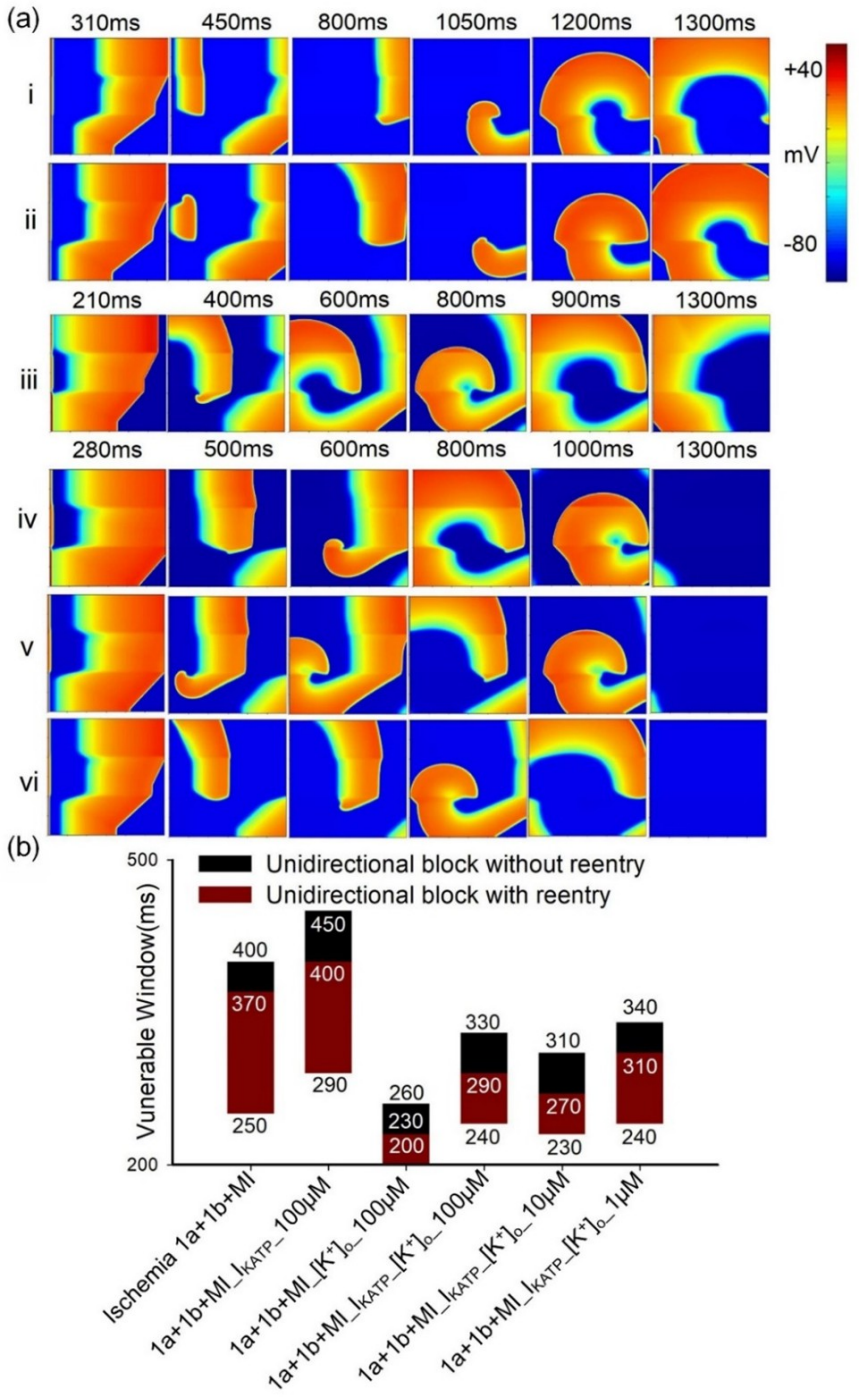
The impact of glibenclamide on excitation wave propagation in 2D heterogeneous tissue using the leftmost fast pacing stimulation protocol. (a) Electrical excitation wave patterns in tissues featuring multiple ischemic conditions with and without the administration of glibenclamide. Specifically, (i) without the administration of glibenclamide; (ii) 1a+1b+MI_I_KATP__100μM; (iii) 1a+1b+MI_[K^+^]_o__100μM; (iv) 1a+1b+MI_I_KATP__[K^+^]_o__100μM; (v) 1a+1b+MI_I_KATP__[K^+^]_o__10μM; (vi) 1a+1b+MI_I_KATP__[K^+^]_o__1μM. 1a+1b+MI_I_KATP__100μM and 1a+1b+MI_[K^+^]_o__100μM represent altering [K^+^]_o_ and I_KATP_ individually during the administration of 100μM glibenclamide. 1a+1b+MI_I_KATP__[K^+^]_o__100μM, 1a+1b+MI_I_KATP__[K^+^]_o__10μM, and 1a+1b+MI_I_KATP__[K^+^]_o__1μM denote simultaneous alterations in [K^+^]_o_ and I_KATP_ during the administration of 100, 10, and 1μM glibenclamide, respectively. (b) Variations in VW in these tissues before and after treatment with glibenclamide.

The underlying mechanisms responsible for the antiarrhythmic effects of glibenclamide are further investigated by manipulating [K^+^]_o_ and I_KATP_ both individually and simultaneously (Fig 8). Since the S1-S2 stimulation protocol is rarely observed in in-vivo circumstances, the leftmost stimulation protocol is employed to simulate the scenario of fast pacing-induced reentrant waves, as described in Liang *et al*. [2]. The results demonstrate that rapid leftmost pacing induces reentrant waves in the tissue with multiple coexisting ischemic conditions (Fig 8A i). When only [K^+^]_o_ is altered in the presence of 100 μM glibenclamide, a longer wavelength (Fig 8A ii) is observed compared to altering only I_KATP_ (Fig 8A iii), suggesting a more profound antiarrhythmic effect of modifying [K^+^]_o_. Moreover, modifying [K^+^]_o_ and I_KATP_ simultaneously has more pronounced effects on suppressing the generation of reentrant waves compared with changing [K^+^]_o_ or I_KATP_ individually (Fig 8A iv-vi). Furthermore, changing [K^+^]_o_ led to a significant reduction in the width of the VW, while altering I_KATP_ had minimal effect on VW width (Fig 8B). The dosage dependence of glibenclamide’s effects on VWs is similar to previous simulation results on tissue with a single ischemic condition (Figs 6B and 8B). These findings provide insights into the specific mechanisms underlying the antiarrhythmic effects of glibenclamide and highlight the importance of [K^+^]_o_ in modulating cardiac electrophysiology.

### The effect of glibenclamide in 3D tissues

**Fig 9:**
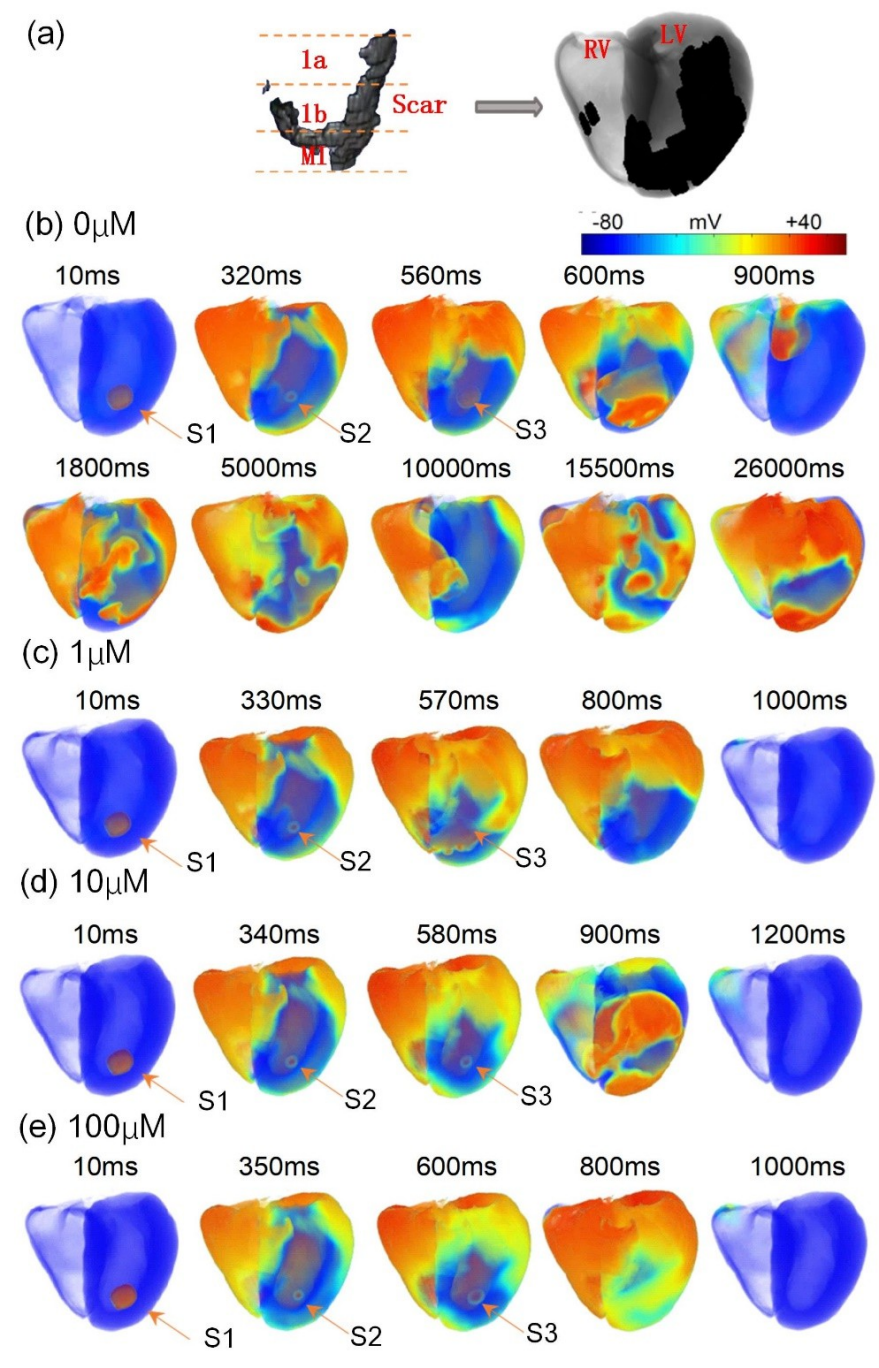
The impact of glibenclamide on excitation wave propagation in the anatomically detailed and realistic models of 3D human ventricular tissue. (a) The 3D real ventricular tissue model depicting the distribution of different ischemic conditions within a scar. (b-e) Electrical excitation wave patterns in the 3D tissues under various conditions: without glibenclamide (b), with the administration of 1 μM (c), 10 μM (d), and 100 μM (e) glibenclamide.

The 3D ventricular tissue model, incorporating multiple ischemic conditions based on a previous study [2] (Fig 9), illustrates the segmentation of the scar into ischemia 1a, 1b, and AMI regions from top to bottom, alongside normal tissues (Fig 9A). This model demonstrates persistent reentry waves within the 3D tissue due to the coexistence of various pathological conditions (Fig 9B). Fig 9C–9E showcases the effects of different concentrations of glibenclamide on reentry wave propagation using a consistent stimulation protocol. The simulation results show that varying concentrations of glibenclamide can inhibit reentrant waves. Remarkably, complete abolishment of reentrant waves is observed with 100 and 1 μM glibenclamide application (Fig 9C and 9E). Although 10 μM glibenclamide doesn’t eliminate reentrant waves, it significantly reduces their maintenance time (Fig 9D). These results strongly support the potent inhibitory effect of glibenclamide on reentry wave occurrence in 3D ventricular tissues.

## Discussion

This study utilizes computational modeling to systematically simulate the therapeutic effects of agents targeting ischemia-induced arrhythmias and to elucidate their underlying mechanisms. Through a comprehensive analysis of electrophysiological remodeling in ischemia and myocardial infarction, several potential antiarrhythmic drugs targeting different factors are identified. Employing multi-scale simulations, this study illuminates the antiarrhythmic mechanism during acute cardiac ischemia, suggesting that pro-arrhythmogenic alterations in cardiac tissue excitability and conduction properties are more significantly influenced by electrophysiological changes in depolarization rate rather than variations in APD. Particularly noteworthy is the remarkable antiarrhythmic effect of glibenclamide, primarily attributed to its role in suppressing potassium ion efflux, thereby facilitating the restitution of [K^+^]_o_, as opposed to the recovery of I_KATP_ during myocardial ischemia. These findings provide specific guidance on potentially effective targets for investigating ischemic arrhythmia. To further validate the proposed hypothesis, comprehensive animal or clinical experiments are necessary to determine their antiarrhythmic efficacy in acute coronary syndrome.

### The antiarrhythmic mechanism of different agents

The formation of a reentrant wave requires the excitation wave’s wavelength (CV multiplied by APD) to be shorter than the conduction path length, as indicated by [31]. An increase in APD or CV impedes reentrant wave generation, thereby reducing the width of VW. Simulation results demonstrate amiodarone’s effective APD prolongation in normal cells but its inefficacy in myocardial cells during acute ischemia. Consequently, it lacks efficacy in treating ischemic arrhythmias. The simulation outcomes highlight the potential hazards of administering amiodarone in circumstances where there is remodeling of calcium and potassium channels due to ischemia. E-4031 and Chromanol 293B primarily inhibit I_Kr_ and I_Ks_, respectively, prolonging action potential duration (APD) and exerting anti-arrhythmic effects. Their combined action significantly reduces the vulnerable window causing reentry in ischemia 1a pathological tissue. Notably, lower drug concentrations induce a smaller vulnerable window (120 ms) compared to higher concentrations (140 ms). This may be attributed to the fact that higher drug concentrations, while extending the APD of cells, lead to a marginal reduction in the maximum depolarization rate of cells (Figs 3 and S1). Acting as an I_Na_ agonist, telmisartan augments the maximum depolarization rate in ischemic cell depolarization phases, thereby enhancing electrical excitation wave conduction velocity in tissue, achieving anti-arrhythmic effects.

In contrast, glibenclamide, by concurrently lowering [K^+^]_o_ and inhibiting I_KATP_, effectively increases both CV and APD. Simulation results on single cells demonstrate that a reduction in [K^+^]_o_ attenuates the inhibitory effect on I_Na_ during ischemia, which aligns with prior findings [32]. Since the variation in I_Na_ is consistent with the trend of CV change as shown in S4 Fig, this reduction of [K^+^]_o_ augments I_Na_, thereby increasing dV/dt_max_ and subsequently elevating CV. Conversely, a reduction in I_KATP_ alone leads to an increase in APD. Hence, the simultaneous reduction in [K^+^]_o_ and I_KATP_ is postulated to extend the wavelength, reduce the VW, and consequently impede the generation of reentrant waves. Intriguingly, simulations on 2D tissue with a single pathological condition reveals that reducing I_KATP_ alone has a minimal effect on VW size, whereas the restoration of [K^+^]_o_ substantially reduces VW size (Fig 8B). Analysis of simulation results on single cells in ischemic 1a after treatment with 100 μM glibenclamide reveal that the increase ratio in APD resulting from decreasing I_KATP_ (approximately a 15% increase relative to APD under the normal condition) is significantly lower than that in CV caused by reducing [K^+^]_o_ (approximately a 65% increase relative to CV under the normal condition), as illustrated in Fig 5B and 5C. Additionally, it’s noted that altering solely [K^+^]_o_ (Fig 8A ii) results in a longer excitation wave wavelength compared to altering only I_KATP_ (Fig 8A iii) at 100 μM glibenclamide. In summary, the recovery ratio of APD is considerably lower than that of CV. Therefore, the solitary decrease in I_KATP_ slightly diminishes VW size due to increased APD, whereas the exclusive restoration of [K^+^]_o_ significantly reduces VW size.

### The effect of glibenclamide on reentry waves in multiple pathological tissues

In the S1-S2 cross-field stimulation protocol, the restoration of [K^+^]_o_ alone markedly reduces the vulnerable window in tissues with single pathological conditions, contributing to the overall reduction of VW observed in multiple pathological tissues. This is consistent with the prior research highlighting that the VW in multiple pathological tissues is shaped by the boundaries set in individual pathological tissues [2]. Moreover, during leftmost side stimulation in tissues affected by multiple ischemic conditions, the exclusive recovery of [K^+^]_o_ amplifies CV values in the three pathological regions, thereby reducing the CV discrepancy between ischemic 1b and MI regions (as depicted in S4B Fig). This action restricts the formation of reentrant waves in the MI area, subsequently minimizing the VW size.

From the perspective of its impact on APD and CV restitution, glibenclamide effectively elevates the APD and CV restitution curves during ischemia 1a, 1b, and short-term MI stages. Additionally, compared to other drugs, the influence of glibenclamide on APD and CV restitution curves in normal cells is minimal, contributing to a reduction in spatial APD and CV dispersion (as depicted in S5 and S6 Figs). However, upon applying leftmost stimulation to multiple pathological tissues, the APD difference exhibits minimal contribution to reentry within the 2D tissues impacted by ischemia and MI. Consequently, the alteration in APD due to the restoration of I_KATP_ alone has limited impact on the VW (Fig 8B).

**Fig 10:**
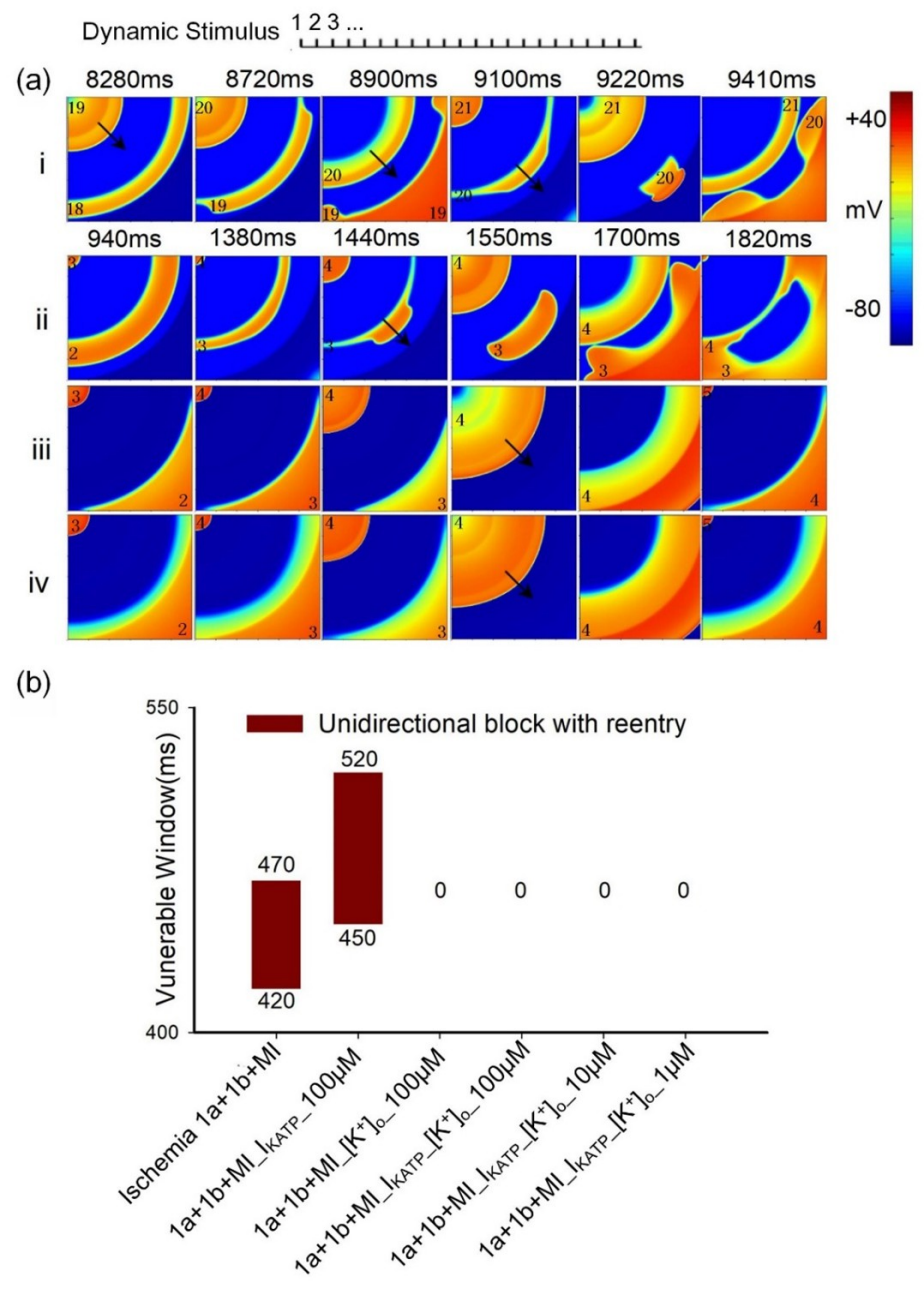
The impact of glibenclamide on excitation wave propagation in 2D heterogeneous tissue featuring an annular distribution of ischemia 1a, 1b, and AMI using upper left corner point stimulation [2]. (a) The electrical excitation wave patterns and (b) variations in VW under various conditions: (i) no glibenclamide, (ii) altering I_KATP_ alone, (iii) changing [K^+^]_o_ alone, and (iv) modifying I_KATP_ and [K^+^]_o_ simultaneously during the application of 100μM glibenclamide. The numbers within the 2D contour plots represent the sequence of stimulations.

Moreover, in accordance with previous findings [2], the heightened excitation threshold in the MI area establishes a barrier effect at the boundary between ischemia 1b and MI regions (Fig 10), potentially fostering the initiation of reentrant waves. Simulation results demonstrate that when I_KATP_ changes alone, the MI region necessitates a high stimulation threshold for excitation, maintaining the barrier effect. However, with a decrease in [K^+^]_o_, the inhibitory effect on I_Na_ weakens, facilitating excitation within the MI region (Fig 10A). Consequently, there is no unidirectional block at the tissue boundary, preventing the emergence of reentrant waves, as illustrated in Fig 10B. Under these circumstances, reentrant waves could be triggered when I_KATP_ is alteration alone, but are notably absent when [K^+^]_o_ decrease.

### The effect of glibenclamide at different concentrations

In the single-cell analysis illustrated in Fig 5, different concentrations of glibenclamide exhibit distinct impacts, most pronounced at 100 μM, followed by 10 μM, and least noticeable at 1 μM. Across 2D tissue models using diverse stimulation protocols, VWs are generally smallest at 100 μM glibenclamide, while they are largest at 1 μM. However, the difference in VWs between 100 μM and 10 μM is marginal. Consequently, the anti-arrhythmic effects of glibenclamide at 100 μM and 10 μM are comparable, both surpassing those at 1 μM. Glibenclamide demonstrates the ability to mitigate spatial electrophysiological heterogeneity in 3D tissues, effectively suppressing the initiation and sustenance of reentrant waves, in line with the observations of Venkatesh et al. [22]. Counterintuitively, 1 μM glibenclamide prevents reentrant wave generation in 3D tissues, while 10 μM glibenclamide does not fully abolish reentry (Fig 9C). However, these outcomes may not be extrapolated to other 3D tissues with differing scar structures, as the formation of reentrant waves in 3D tissues is intricately linked to scar size and position [33]. The dosage-dependent effects of glibenclamide on VWs within 3D tissue do not align consistently with the simulation outcomes observed in single cells and on 2D tissue, implying a considerably more complex mechanism for the genesis of reentrant waves in the 3D heart. Furthermore, the administration of high concentrations of glibenclamide poses the risk of hypoglycemia [34]. Therefore, determining the optimal concentration of glibenclamide for antiarrhythmic purposes on 3D cardiac tissue necessitates further physiological experiments and more detailed computational models that incorporate cardiac metabolism.

## Limitations

Firstly, this study utilizes the TP06 cell model at the single-cell level, encompassing the limitations of the TP06 model. Subsequently, the 2D tissue models employed in this study are idealized representations, potentially subject to validation using real cardiac tissues in future studies, yet such validation wouldn’t alter our conclusions. Furthermore, the devised distribution pattern of ischemic and MI tissues in this research is idealized, and future investigations may require more realistic distribution patterns for ischemic and MI tissues. However, these limitations do not compromise our conclusions regarding the antiarrhythmic mechanism of agents in this study. Further validation of our findings could be facilitated by employing additional 3D models with diverse scar structures. Furthermore, our study utilizes three-dimensional tissue models consisting of isotropic domains, neglecting consideration of fiber orientation. To investigate the impact of anisotropy, it is essential to develop a model that incorporates fiber direction, potentially through the utilization of a rule-based Laplace-Dirichlet fiber direction generation algorithm.

Notably, the agents examined in this study consistently reduced VW sizes but did not entirely prevent reentry formation. Subsequent research will explore whether a combination of drugs targeting multiple therapeutic targets yields superior antiarrhythmic effects. Moreover, this study measures the efficacy of an antiarrhythmic drug primarily based on the alteration in the size of the vulnerable window. However, drugs whose effects decrease the vulnerable window under a specific set of assumptions may promote arrhythmias under alternative conditions, as observed in the CAST trial. Hence, while this study provides insights into the potential antiarrhythmic properties of the drugs, more comprehensive animal or clinical experiments are still imperative to determine their clinical efficacy.

In this study, the ischemic remodeling primarily focuses on electrophysiological remodeling, neglecting mechanical remodeling. Mechanical remodeling has the potential to affect the dynamics of ion channels and myocardial fiber structure, among other factors. Future models should incorporate the potential effects of mechanics, especially mechanical heterogeneity during regional ischemia, on electrophysiology. Moreover, electrophysiological characteristics may vary from one beat to the next, as well as from one cell to another. In the future, more complex electrophysiological remodeling, such as cardiac alternans, and various types of cell models, such as Purkinje fiber networks and nodal systems, should be incorporated.

## Supporting information

S1 Fig Variations in electrophysiological characteristics of cardiomyocytes under different conditions: normal, ischemia 1a, ischemia 1a treated with 30nM E-4031, 81nM E-4031, 3.5μM Chromanol 293B, 10.4μM Chromanol 293B, a combination of 30nM E-4031 and 3.5μM Chromanol 293B, and a combination of 81nM E-4031 and 10.4μM Chromanol 293B. (a)AP profiles (b)CVs (c) AP characteristics, including APA, RP, APD_90_, and dV/dt_max_.
(TIF)

S2 Fig Variations in electrophysiological characteristics of cardiomyocytes under different conditions: normal, ischemia 1a, and ischemia 1a with the application of 0.1μM, 1.2μM, and 5.5μM telmisartan. (a)AP profiles (b)CVs (c) AP characteristics, including APA, RP, APD90, and dV/dtmax.
(TIF)

S3 Fig Variations in the vulnerable window (VW) in different tissues: (a) ischemia 1b without cell decoupling, (b) MI without cell decoupling, (c) decoupled ischemia 1b, and (d) decoupled MI tissues before and after treatment with 100μM, 10μM, and 1μM glibenclamide. Specifically, the effects of altering only [K^+^]_o_ or I_KATP_ during the administration of 100μM glibenclamide are included.
(TIF)

S4 Fig The normalized alterations in (a) I_Na_ and (b) CV observed in ischemic conditions (1a, 1b, and MI), presented as values normalized against those of ischemia 1b, both with and without glibenclamide treatment.
(TIF)

S5 Fig The effect of the different drugs on APD restitution. (a) without drugs, (b) amiodarone, (c) E-4031 and Chromanol 293B, (d) telmisartan and (e) glibenclamide.
(TIF)

S6 Fig The effect of the different drugs on CV restitution. (a) without drugs, (b) amiodarone, (c) E-4031 and Chromanol 293B, (d) telmisartan and (e) glibenclamide.
(TIF)

## References

[1] Anversa P, Li P, Zhang X, Olivetti G, Capasso JM. Ischaemic myocardial injury and ventricular remodelling. Cardiovasc Res. 1993 Feb;27(2):145–57. doi: 10.1093/cvr/27.2.145 . 8472264

[2] Liang C, Li Q, Wang K, Du Y, Wang W, Zhang H. Mechanisms of ventricular arrhythmias elicited by coexistence of multiple electrophysiological remodeling in ischemia: A simulation study. PLoS Comput Biol. 2022 Apr 27;18(4):e1009388. doi: 10.1371/journal.pcbi.1009388 ; PMCID: PMC9045648. 35476614 PMC9045648

[3] Trayanova NA, Lyon A, Shade J, Heijman J. Computational modeling of cardiac electrophysiology and arrhythmogenesis. Physiol Rev. 2023 Dec 28. doi: 10.1152/physrev.00017.2023 Epub ahead of print. . 38153307 PMC11381036

[4] Dutta S, Mincholé A, Quinn TA, Rodriguez B. Electrophysiological properties of computational human ventricular cell action potential models under acute ischemic conditions. Prog Biophys Mol Biol. 2017 Oct;129:40–52. doi: 10.1016/j.pbiomolbio.2017.02.007 Epub 2017 Feb 20. . 28223156

[5] Kazbanov IV, Clayton RH, Nash MP, Bradley CP, Paterson DJ, Hayward MP, Taggart P, Panfilov AV. Effect of global cardiac ischemia on human ventricular fibrillation: insights from a multi-scale mechanistic model of the human heart. PLoS Comput Biol. 2014 Nov 6;10(11):e1003891. doi: 10.1371/journal.pcbi.1003891 ; PMCID: PMC4222598. 25375999 PMC4222598

[6] Clayton RH, Nash MP, Bradley CP, Panfilov AV, Paterson DJ, Taggart P. Experiment-model interaction for analysis of epicardial activation during human ventricular fibrillation with global myocardial ischaemia. Prog Biophys Mol Biol. 2011 Oct;107(1):101–11. doi: 10.1016/j.pbiomolbio.2011.06.010 Epub 2011 Jul 2. . 21741985

[7] De Groot JR, Coronel R. Acute ischemia-induced gap junctional uncoupling and arrhythmogenesis. Cardiovasc Res. 2004 May 1;62(2):323–34. doi: 10.1016/j.cardiores.2004.01.033 . 15094352

[8] Alday EA, Ni H, Zhang C, Colman MA, Gan Z, Zhang H. Comparison of Electric- and Magnetic-Cardiograms Produced by Myocardial Ischemia in Models of the Human Ventricle and Torso. PLoS One. 2016 Aug 24;11(8):e0160999. doi: 10.1371/journal.pone.0160999 ; PMCID: PMC4996509. 27556808 PMC4996509

[9] Smith WT 4th, Fleet WF, Johnson TA, Engle CL, Cascio WE. The Ib phase of ventricular arrhythmias in ischemic in situ porcine heart is related to changes in cell-to-cell electrical coupling. Experimental Cardiology Group, University of North Carolina. Circulation. 1995 Nov 15;92(10):3051–60. doi: 10.1161/01.cir.92.10.3051 . 7586276

[10] Jongsma HJ, Wilders R. Gap junctions in cardiovascular disease. Circ Res. 2000 Jun 23;86(12):1193–7. doi: 10.1161/01.res.86.12.1193 . 10864907

[11] Lei M, Wu L, Terrar DA, Huang CL. Modernized Classification of Cardiac Antiarrhythmic Drugs. Circulation. 2018 Oct 23;138(17):1879–1896. doi: 10.1161/CIRCULATIONAHA.118.035455 Erratum in: Circulation. 2019 Mar 26;139(13):e635. . 30354657

[12] Kamiya K, Nishiyama A, Yasui K, Hojo M, Sanguinetti MC, Kodama I. Short- and long-term effects of amiodarone on the two components of cardiac delayed rectifier K(+) current. Circulation. 2001 Mar 6;103(9):1317–24. doi: 10.1161/01.cir.103.9.1317 . 11238279

[13] McPate MJ, Duncan RS, Witchel HJ, Hancox JC. Disopyramide is an effective inhibitor of mutant HERG K+ channels involved in variant 1 short QT syndrome. J Mol Cell Cardiol. 2006 Sep;41(3):563–6. doi: 10.1016/j.yjmcc.2006.05.021 Epub 2006 Jul 12. . 16842817

[14] Sun ZQ, Thomas GP, Antzelevitch C. Chromanol 293B inhibits slowly activating delayed rectifier and transient outward currents in canine left ventricular myocytes. J Cardiovasc Electrophysiol. 2001 Apr;12(4):472–8. doi: 10.1046/j.1540-8167.2001.00472.x . 11332571

[15] Yusuf S, Diener HC, Sacco RL, Cotton D, Ounpuu S, Lawton WA, Palesch Y, Martin RH, Albers GW, Bath P, Bornstein N, Chan BP, Chen ST, Cunha L, Dahlöf B, De Keyser J, Donnan GA, Estol C, Gorelick P, Gu V, Hermansson K, Hilbrich L, Kaste M, Lu C, Machnig T, Pais P, Roberts R, Skvortsova V, Teal P, Toni D, VanderMaelen C, Voigt T, Weber M, Yoon BW; PRoFESS Study Group. Telmisartan to prevent recurrent stroke and cardiovascular events. N Engl J Med. 2008 Sep 18;359(12):1225–37. doi: 10.1056/NEJMoa0804593 Epub 2008 Aug 27. ; PMCID: PMC2714258. 18753639 PMC2714258

[16] Chang TT, Yang CJ, Lee YC, Wu SN. Stimulatory Action of Telmisartan, an Antagonist of Angiotensin II Receptor, on Voltage-Gated Na+ Current: Experimental and Theoretical Studies. Chin J Physiol. 2018 Feb 28;61(1):1–13. doi: 10.4077/CJP.2018.BAG516 . 29374954

[17] Luzi L, Pozza G. Glibenclamide: an old drug with a novel mechanism of action? Acta Diabetol. 1997 Dec;34(4):239–44. doi: 10.1007/s005920050081 . 9451465

[18] Lomuscio A, Vergani D, Marano L, Castagnone M, Fiorentini C. Effects of glibenclamide on ventricular fibrillation in non-insulin-dependent diabetics with acute myocardial infarction. Coron Artery Dis. 1994 Sep;5(9):767–71. . 7858767

[19] El-Reyani NE, Baczkó I, Leprán I, Papp JG. Effect of glibenclamide and glimepiride treatment on the development of myocardial infarction in rats. Acta Physiol Hung. 2000;87(2):173–84. . 11205966

[20] Cacciapuoti F, Spiezia R, Bianchi U, Lama D, D’Avino M, Varricchio M. Effectiveness of glibenclamide on myocardial ischemic ventricular arrhythmias in non-insulin-dependent diabetes mellitus. Am J Cardiol. 1991 Apr 15;67(9):843–7. doi: 10.1016/0002-9149(91)90617-t . 1707221

[21] Cole WC, McPherson CD, Sontag D. ATP-regulated K+ channels protect the myocardium against ischemia/reperfusion damage. Circ Res. 1991 Sep;69(3):571–81. doi: 10.1161/01.res.69.3.571 . 1908354

[22] Venkatesh N, Lamp ST, Weiss JN. Sulfonylureas, ATP-sensitive K+ channels, and cellular K+ loss during hypoxia, ischemia, and metabolic inhibition in mammalian ventricle. Circ Res. 1991 Sep;69(3):623–37. doi: 10.1161/01.res.69.3.623 . 1908355

[23] Wilde AA, Escande D, Schumacher CA, Thuringer D, Mestre M, Fiolet JW, Janse MJ. Potassium accumulation in the globally ischemic mammalian heart. A role for the ATP-sensitive potassium channel. Circ Res. 1990 Oct;67(4):835–43. doi: 10.1161/01.res.67.4.835 . 2119912

[24] Taggart P, Sutton PM, Opthof T, Coronel R, Trimlett R, Pugsley W, Kallis P. Inhomogeneous transmural conduction during early ischaemia in patients with coronary artery disease. J Mol Cell Cardiol. 2000 Apr;32(4):621–30. doi: 10.1006/jmcc.2000.1105 . 10756118

[25] Brennan T, Fink M, Rodriguez B. Multiscale modelling of drug-induced effects on cardiac electrophysiological activity. Eur J Pharm Sci. 2009 Jan 31;36(1):62–77. doi: 10.1016/j.ejps.2008.09.013 Epub 2008 Nov 17. . 19061955

[26] Lalevée N, Nargeot J, Barrére-Lemaire S, Gautier P, Richard S. Effects of amiodarone and dronedarone on voltage-dependent sodium current in human cardiomyocytes. J Cardiovasc Electrophysiol. 2003 Aug;14(8):885–90. doi: 10.1046/j.1540-8167.2003.03064.x . 12890054

[27] Gray DF, Mihailidou AS, Hansen PS, Buhagiar KA, Bewick NL, Rasmussen HH, Whalley DW. Amiodarone inhibits the Na(+)-K+ pump in rabbit cardiac myocytes after acute and chronic treatment. J Pharmacol Exp Ther. 1998 Jan;284(1):75–82. . 9435163

[28] Nishimura M, Follmer CH, Singer DH. Amiodarone blocks calcium current in single guinea pig ventricular myocytes. J Pharmacol Exp Ther. 1989 Nov;251(2):650–9. . 2553932

[29] Watanabe Y, Kimura J. Acute inhibitory effect of dronedarone, a noniodinated benzofuran analogue of amiodarone, on Na+/Ca2+ exchange current in guinea pig cardiac ventricular myocytes. Naunyn Schmiedebergs Arch Pharmacol. 2008 Jun;377(4–6):371–6. doi: 10.1007/s00210-008-0270-2 Epub 2008 Apr 8. . 18392809

[30] Zankov DP, Ding WG, Matsuura H, Horie M. Open-state unblock characterizes acute inhibition of I potassium current by amiodarone in guinea pig ventricular myocytes. J Cardiovasc Electrophysiol. 2005 Mar;16(3):314–22. doi: 10.1046/j.1540-8167.2005.40561.x . 15817093

[31] Gaztañaga L, Marchlinski FE, Betensky BP. Mechanisms of cardiac arrhythmias. Rev Esp Cardiol (Engl Ed). 2012 Feb;65(2):174–85. English, Spanish. doi: 10.1016/j.recesp.2011.09.018 Epub 2011 Dec 21. . 22192903

[32] Whalley DW, Wendt DJ, Starmer CF, Rudy Y, Grant AO. Voltage-independent effects of extracellular K+ on the Na+ current and phase 0 of the action potential in isolated cardiac myocytes. Circ Res. 1994 Sep;75(3):491–502. doi: 10.1161/01.res.75.3.491 . 8062422

[33] Liang C, Wang K, Li Q, Bai J, Zhang H. Influence of the distribution of fibrosis within an area of myocardial infarction on wave propagation in ventricular tissue. Sci Rep. 2019 Oct 2;9(1):14151. doi: 10.1038/s41598-019-50478-5 ; PMCID: PMC6775234. 31578428 PMC6775234

[34] Bijlstra PJ, Lutterman JA, Russel FG, Thien T, Smits P. Interaction of sulphonylurea derivatives with vascular ATP-sensitive potassium channels in humans. Diabetologia. 1996 Sep;39(9):1083–90. doi: 10.1007/BF00400658 Erratum in: Diabetologia 1996 Nov;39(11):1414. . 8877293

