# Supplementary figures and images for: A simulation study on the antiarrhythmic mechanisms of established agents in myocardial ischemia and infarction
Source: PLoS Comput Biol. 2024 Jun 25;20(6):e1012244. doi: 10.1371/journal.pcbi.1012244 (PMC11230589; doi:10.1371/journal.pcbi.1012244)

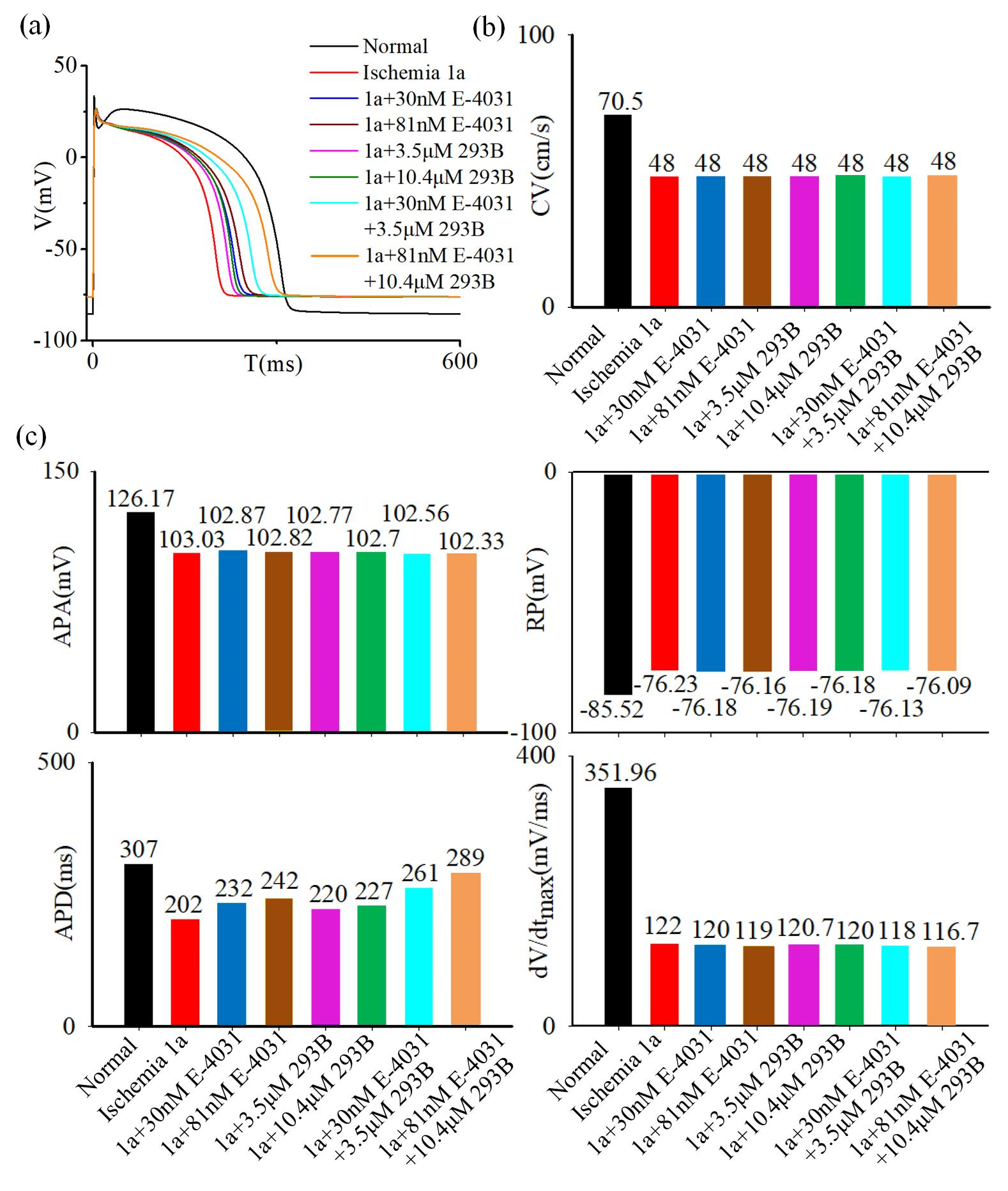

Supplement: S1 Fig — (a)AP profiles (b)CVs (c) AP characteristics, including APA, RP, APD90, and dV/dtmax. (TIF) [file pcbi.1012244.s001.tif]

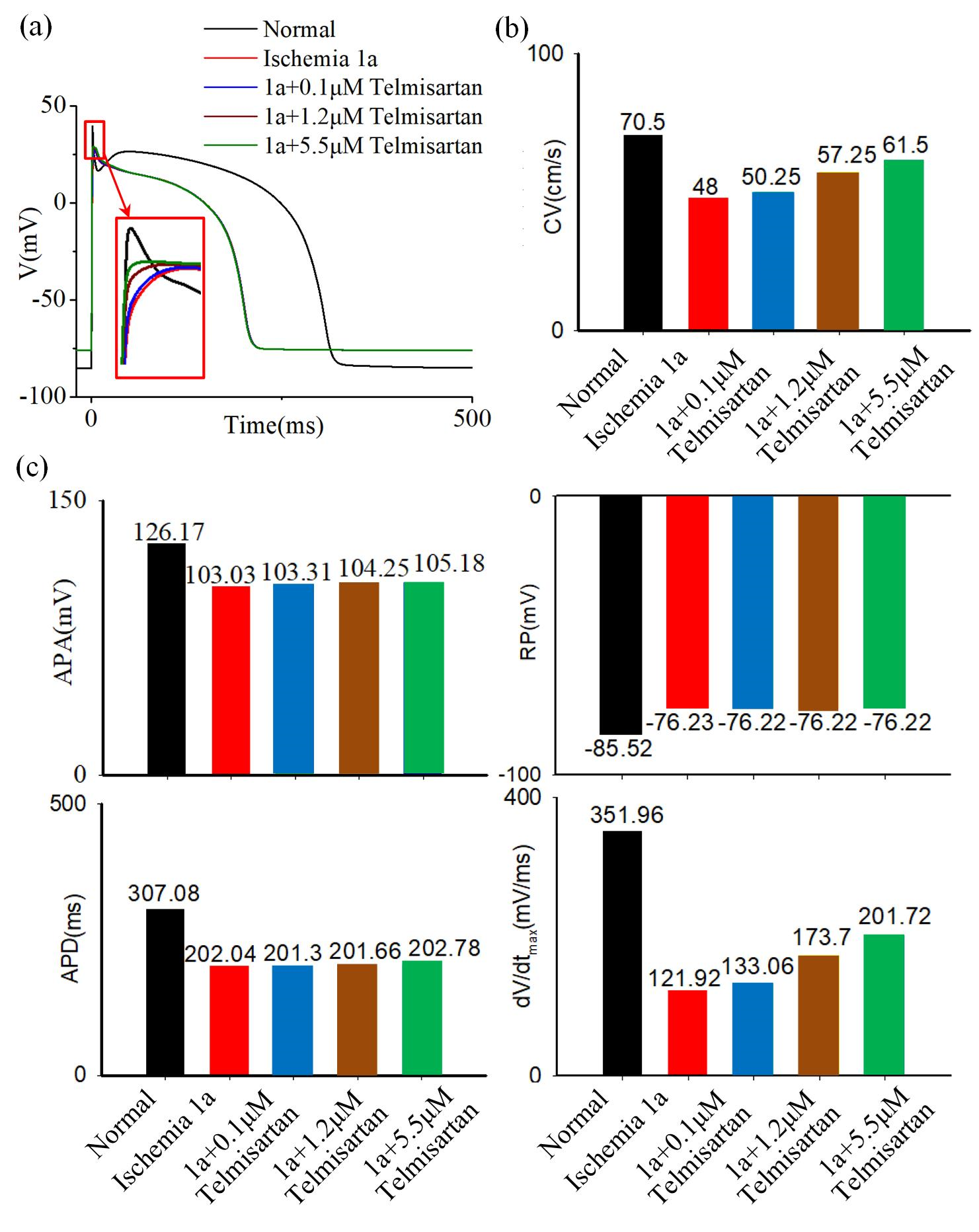

Supplement: S2 Fig — (a)AP profiles (b)CVs (c) AP characteristics, including APA, RP, APD90, and dV/dtmax. (TIF) [file pcbi.1012244.s002.tif]

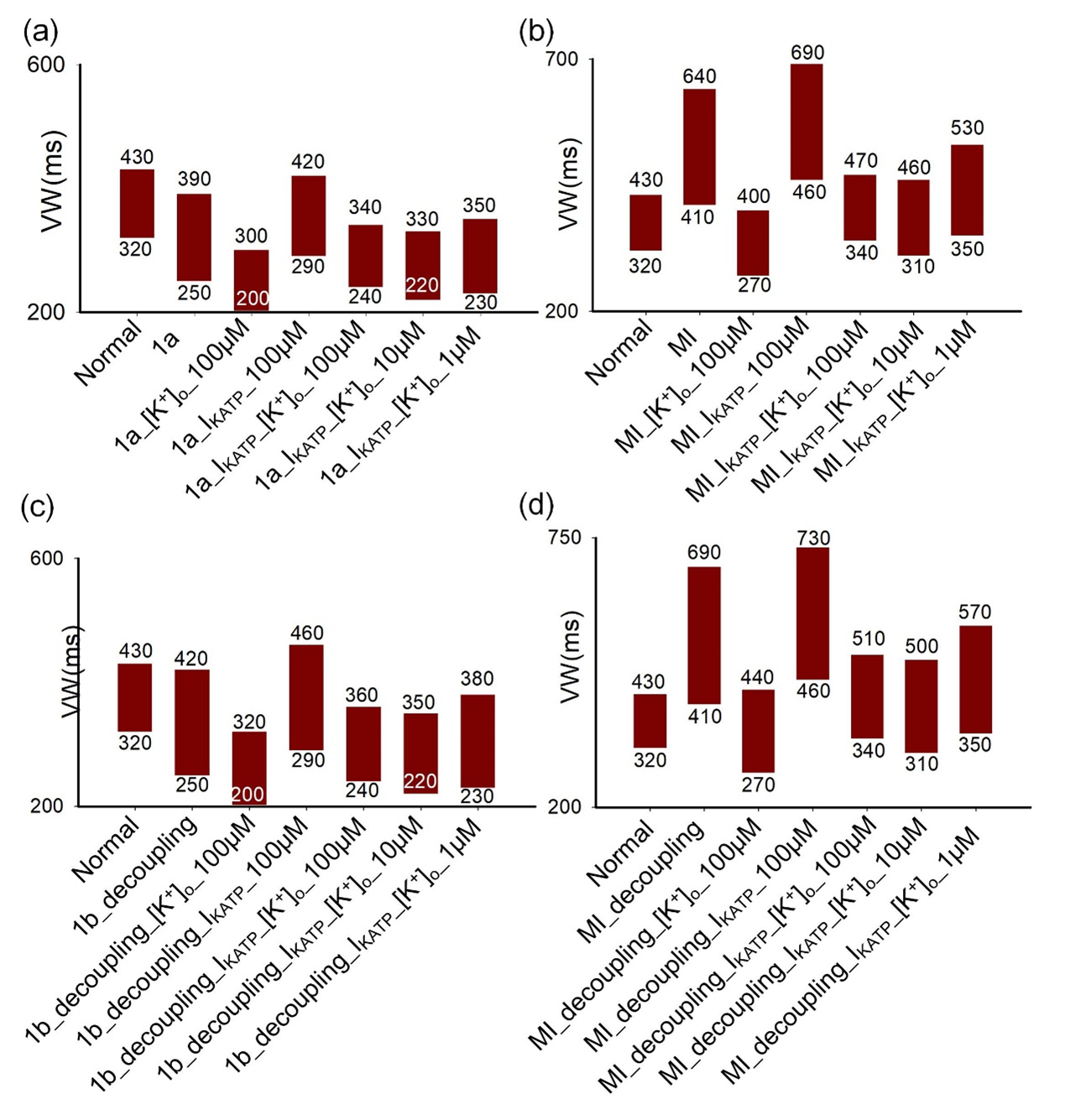

Supplement: S3 Fig — Variations in the vulnerable window (VW) in different tissues: (a) ischemia 1b without cell decoupling, (b) MI without cell decoupling, (c) decoupled ischemia 1b, and (d) decoupled MI tissues before and after treatment with 100μM, 10μM, and 1μM glibenclamide. Specifically, the effects of altering only [K+]o or IKATP during the administration of 100μM glibenclamide are included. (TIF) [file pcbi.1012244.s003.tif]

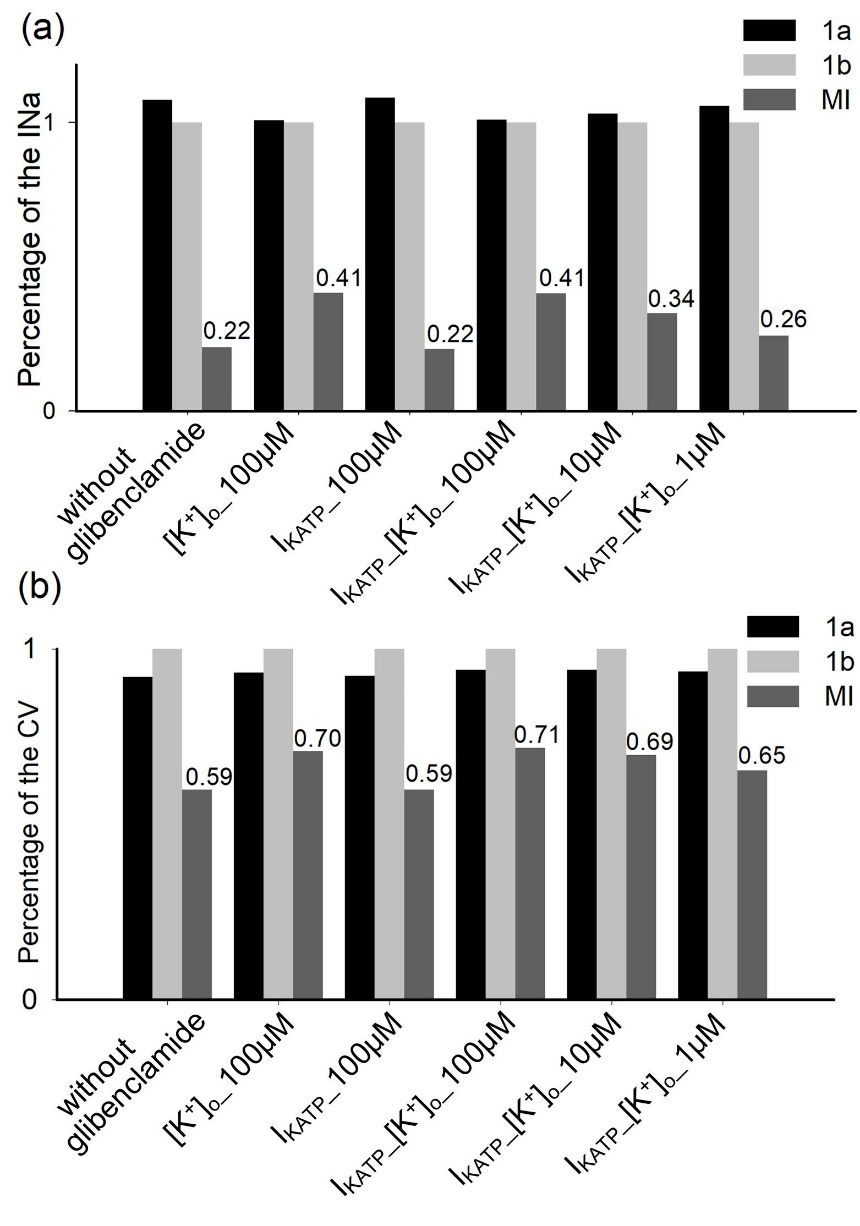

Supplement: S4 Fig — The normalized alterations in (a) INa and (b) CV observed in ischemic conditions (1a, 1b, and MI), presented as values normalized against those of ischemia 1b, both with and without glibenclamide treatment. (TIF) [file pcbi.1012244.s004.tif]

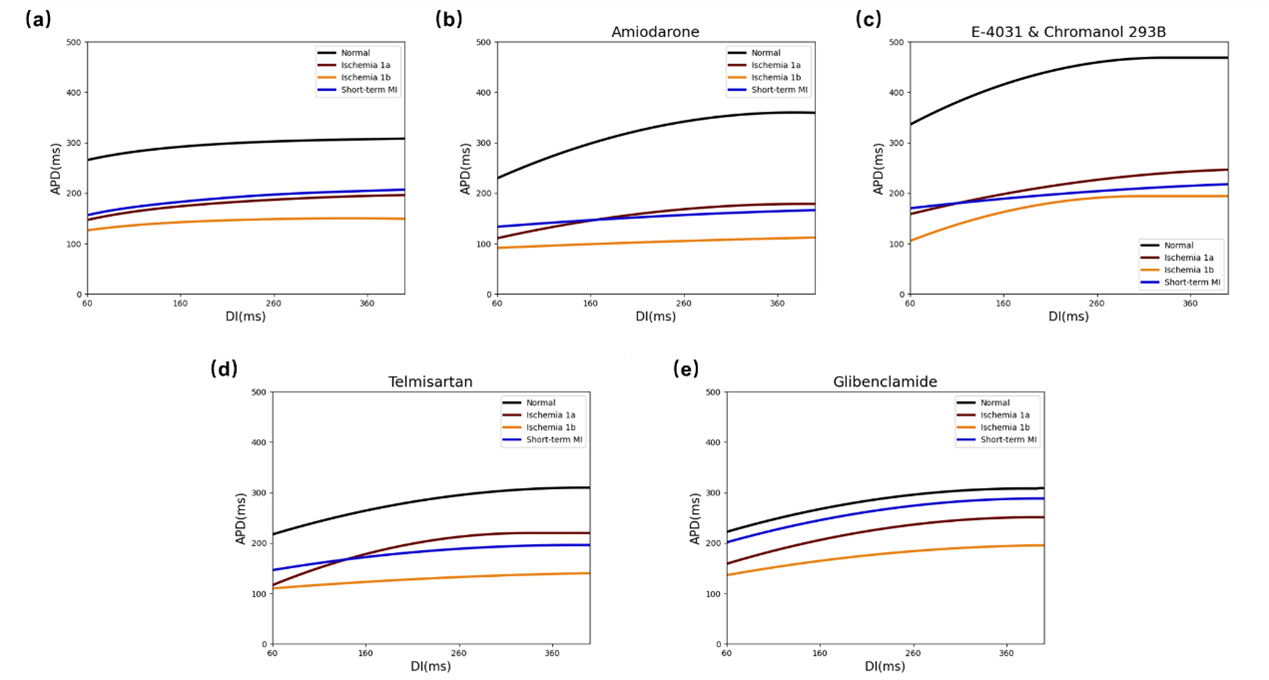

Supplement: S5 Fig — (a) without drugs, (b) amiodarone, (c) E-4031 and Chromanol 293B, (d) telmisartan and (e) glibenclamide. (TIF) [file pcbi.1012244.s005.tif]

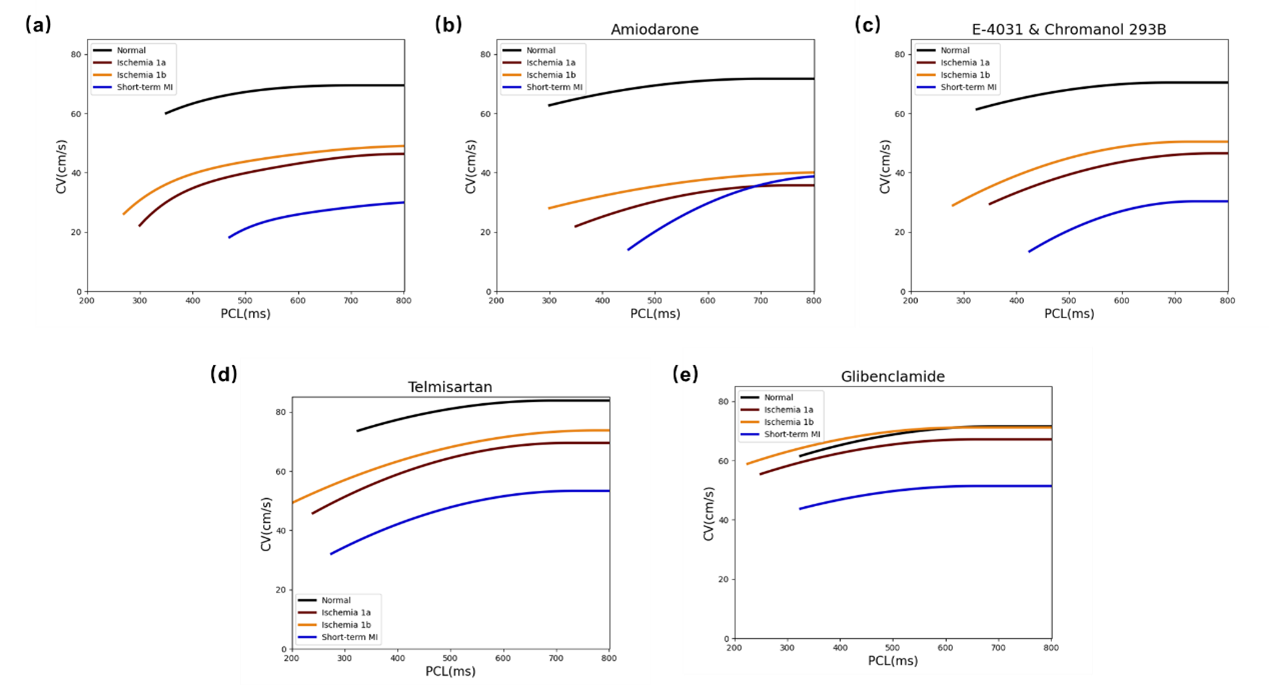

Supplement: S6 Fig — (a) without drugs, (b) amiodarone, (c) E-4031 and Chromanol 293B, (d) telmisartan and (e) glibenclamide. (TIF) [file pcbi.1012244.s006.tif]
